# Atmospheric dispersal shapes rapid bacterial colonization of Icelandic Lava Rocks

**DOI:** 10.1093/femsmc/xtae016

**Published:** 2024-05-24

**Authors:** Aurélien Daussin, Pauline Vannier, Lola Daboussy, Tina Šantl-Temkiv, Charles Cockell, Viggó Þór Marteinsson

**Affiliations:** Faculty of Food Science and Nutrition, University of Iceland, Sæmundargatu 2, 102 Reykjavík, Iceland; MATIS, Department of Research and Innovation, Vinlandsleið 12, 113 Reykjavík, Iceland; MATIS, Department of Research and Innovation, Vinlandsleið 12, 113 Reykjavík, Iceland; Université de Toulon, MAPIEM, SeaTech, Bâtiment X, Avenue de l'Université, 83130 La Garde, France; University of Technology of Compiègne, CS 60319, 60203 Compiègne, France; Department of Biology, Aarhus University, Ny Munkegade 114, 8000 Aarhus, Denmark; Department of Biology, Arctic Research Center, Aarhus University, Ole Worms Allé 1, 8000 Aarhus, Denmark; Department of Environmental Science, iCLIMATE Aarhus University Interdisciplinary Centre for Climate Change, Aarhus University, Ny Munkegade 116, 8000 Aarhus, Denmark; School of Physics and Astronomy, University of Edinburgh, James Clerk Maxwell Building, Peter Guthrie Tait Road, Edinburgh, Scotland; Faculty of Food Science and Nutrition, University of Iceland, Sæmundargatu 2, 102 Reykjavík, Iceland; MATIS, Department of Research and Innovation, Vinlandsleið 12, 113 Reykjavík, Iceland; The Agricultural University of Iceland, Hvanneyri, 311 Borgabyggð, Iceland

**Keywords:** bioaerosols, microbial diversity, amplicon sequencing, volcanic rocks, colonization, source apportionment, Iceland

## Abstract

Microorganisms released into the atmosphere by various disturbances can travel significant distances before depositing, yet their impact on community assembly remains unclear. To address this, we examined atmospheric and lithospheric bacterial communities in 179 samples collected at two distinct Icelandic volcanic sites: a small volcanic island Surtsey, and a volcanic highland Fimmvörðuháls using 16S rRNA amplicon sequencing. Airborne microbial communities were similar between sites while significant differences emerged in the communities on lava rocks after 1-year exposure. SourceTracker analysis revealed distinct bacterial populations in the atmosphere and the lava rocks with surrounding soil contributed more significantly to lava rock microbial composition. Nevertheless, shared genera among air, rocks, and local sources, suggested potential exchange between these environments. The prevalent genera shared between rocks and potential sources exhibited stress-resistant properties, likely helping their survival during air transportation and facilitating their colonization of the rocks. We hypothesize that the atmosphere serves as a conduit for locally sourced microbes and stress-resistant distant-sourced microbes. Additionally, bacterial communities on the lava rocks of Fimmvörðuháls showed remarkable similarity after 1 and 9 years of exposure, suggesting rapid establishment. Our study reveals that atmospheric deposition significantly influences bacterial community formation, potentially influencing ecosystem dynamics and microbial communities’ resilience.

## Teaser

The impact of atmospheric deposition on bacterial community formation remains poorly understood. This study reports that atmospheric dispersal plays a crucial role in colonizing volcanic rocks, leading to the rapid formation of unique and diverse communities within just one year.

## Introduction

Particles of biological origin suspended in the atmosphere are referred to as primary biological aerosol particles (Emetere et al. 2021). These particles are released from the Earth’s surface into the air by wind or physical disturbances such as agricultural activity or wildfires (Fröhlich-Nowoisky et al. 2016, Šantl-Temkiv et al. 2022) and are ubiquitously represented. The density and composition of airborne microbes and communities primarily rely on the source of their emissions, which can be either natural sources like water bodies, soils, and vegetated areas, or anthropogenic sources (Xie et al. 2021).

Microorganisms are usually the first to settle on new land or surfaces, influencing the growth of communities over time (Šantl-Temkiv et al. 2022). The process of colonization is influenced by mineral properties, soil pH, and microbial traits. Notably, bacterial binding to minerals, facilitated by extracellular polysaccharides and proteins, is crucial for stable colonization (Vieira et al. 2020), but is notably reduced in acidic and alkaline soils (Malard and Pearce 2022). Famous for its active volcanoes, Iceland offers an optimal setting to investigate the colonization and evolution of microorganisms on recently created volcanic rocks. On a more localized scale, the volcanic island Surstey, formed in 1963–1967 by an underwater eruption, was one of the first places where researchers began studying this. They noted the presence of phototrophs by 1968 (Sigurdsson 1970). However, the study of the inland Fimmvörðuhals lava field, formed in 2010, showed contrasting results, with the early dominance of diazotrophs, chemolithotrophs, and heterotrophs rather than phototrophs (Kelly et al. 2014). A recent study comparing the atmospheric and lithospheric culturable bacterial communities from Surtsey and Fimmvörðuháls (Daussin et al. 2023b) also revealed that Proteobacteria and Actinobacteria were the dominating phyla. Furthermore, the study discovered notable differences between the microbial communities present in the atmosphere and lithosphere, and the proportion of bacterial taxa isolated from each site was influenced by the location’s environment and characteristics.

Research indicates that the atmosphere is the primary contributor to the dispersion of microbial cells in recently formed environments. Despite this, there is a notable lack of comprehensive studies on the processes of dispersal, colonization, and succession in these areas. Additionally, the role of atmospheric deposition in community assembly remains to be understood. Studying these aspects is crucial for comprehending ecosystem dynamics and contributing to scientific knowledge in the fields of extreme microbiology, ecology, and environmental science.

This study had three main goals: (i) to examine and compare the microbial diversity of air and 1-year-old lava rock samples of two Icelandic volcanic sites—Surtsey Island and Fimmvörðuháls Highland, (ii) to compare the microbial composition of lava rocks at Fimmvörðuháls after 1 and 9 years of exposure to the air, and (iii) to investigate the origin of lava rocks microbes in Surtsey after 1-year exposure to the air. From the 179 samples, around 50 000 filtered amplicon sequence variants (ASVs) or single DNA sequences were used for assessing and comparing the microbial diversity on both sites, using statistical tools. The comparison of microbial communities was used to investigate the origin of the lava rocks in Surtsey.

## Materials and method

### Site description and sample collection

**Figure 1.**
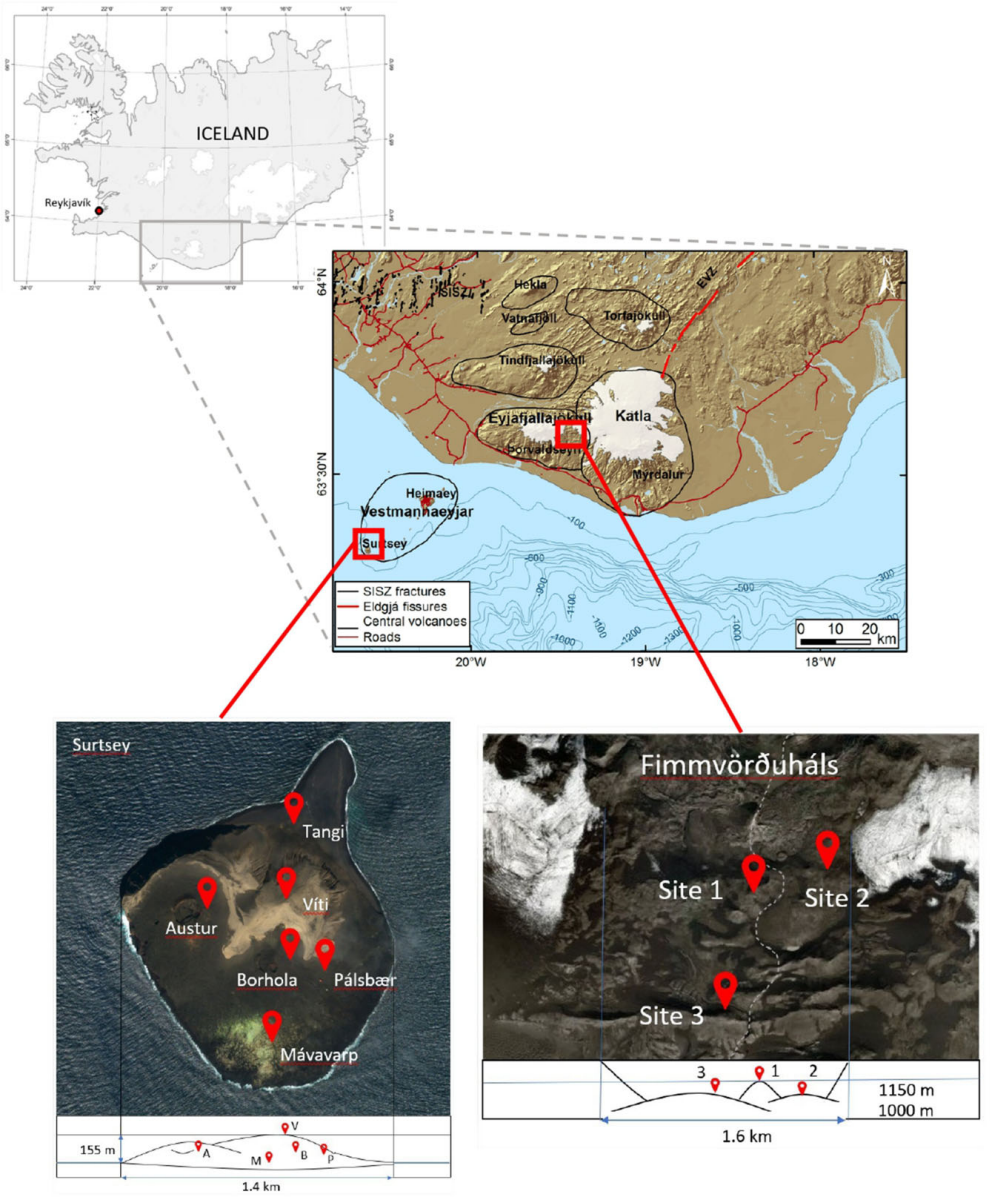
Location of the sampling sites Surtsey and Fimmvörðuháls, and the stations. (Daussin et al. 2023a).

To compare environmental influences on microbial composition and draw hypotheses on the origin and dispersal of microbes, samples were collected from two distinct volcanic sites in Iceland (Daussin et al. 2023b). Briefly, the Surtsey site (63°18′11″N, 20°36′11″W) is a newly formed volcanic island situated off the southern coast of Iceland. Its formation resulted from an underwater volcanic eruption lasting 4 years, from 1963 to 1967. UNESCO declared the island a World Heritage Site and it has been protected since its formation, remaining closed to the general public (UNESCO—World Heritage Convention). The Fimmvörðuháls site (63°37′53″N, 19°26′50″W, altitude 1100 m) is positioned between the Eyjafjallajökull and Mýrdalsjökull glaciers in the southern region of Iceland. This lava field originated from the eruption of the Eyjafjallajökull volcano in the spring of 2010. Each site was divided into different sampling stations, with five stations in Surtsey and three stations in Fimmvörðuháls (Fig. 1). Samples from Surtsey were recovered on 19–22 July 2018 and 18–22 July 2019, and from Fimmvörðuháls on 12–14 September 2018 and 27–28 September 2019. Different types of samples were obtained at each site during each sampling period.

**Figure 2.**
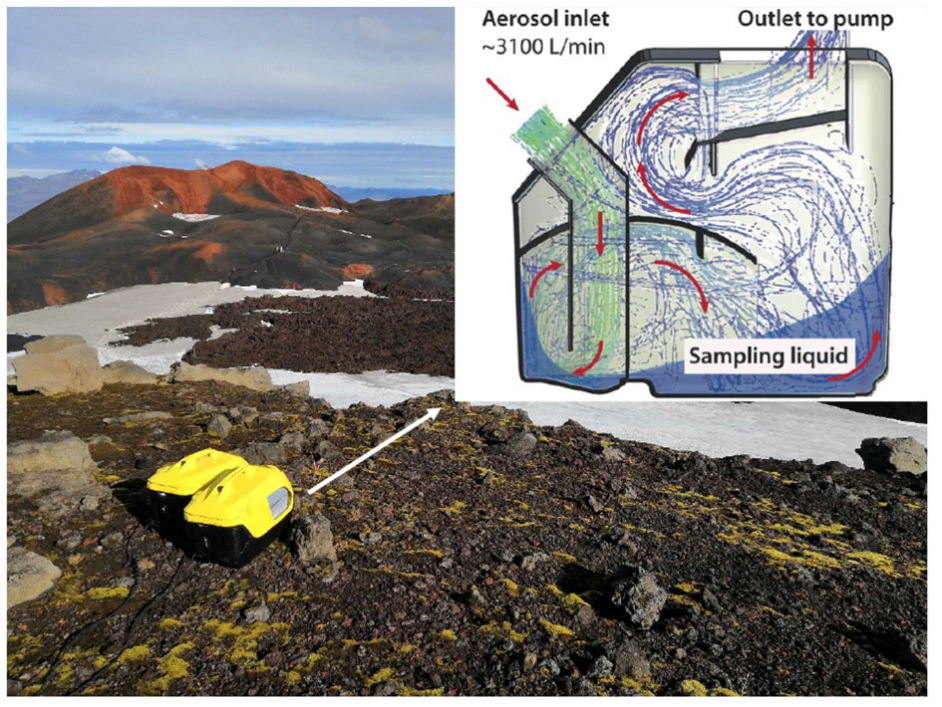
High flow-rate impinger collecting air samples in Fimmvörðuháls in 2018 and schematic view of its sampling process (Šantl-Temkiv et al. 2017).

We used air and lava rock samples previously collected as described in our prior publication (Daussin et al. 2023b). Briefly, we employed high-flow-rate impingers (Fig. 2) for air sample collection, and recovered on-site autoclaved lava rocks after 1 year, denoted as “1-year-old lava rocks.” Additionally, nonautoclaved 9-year-old lava rocks from Fimmvörðuháls were designated as “9-years-old lava rocks.” The decision to sample old lava rocks from Fimmvörðuháls was made after the first expedition to Surtsey, where the focus was on collecting samples. This choice was influenced by the greater accessibility of Fimmvörðuháls compared to the remote and challenging access to Surtsey, allowing to study older volcanic rocks in a more feasible manner. The 1-year-old lava rocks were fragmented using a sterilized hammer to facilitate analyses of the top, middle, and bottom parts. Based on the first results and to enhance clarity and align with our goals, the microbial communities from all three sections will be combined and analyzed collectively.

Soil was collected as a potential source of the 1-year-old lava rock microbes in Surtsey in 2019 and kept at −20°C before being stored at −80°C back in the laboratory. It was collected within 10 cm of the sampled rock. Additionally, 16S rRNA sequences from fumarole, drill core, and seawater samples collected on Surtsey by Bergsten et al. (2021) were incorporated into this study to explore potential sources for the 1-year-old lava rocks of Surtsey. The total number of samples, comprising duplicates of air samples with controls and rock samples from various sections, ages, and locations (Surtsey and Fimmvörðuháls), added up to 179. The coordinates, characteristics, number, and type of sample recovered from the different stations can be found in Supplementary Material – Table 1.

### DNA extraction, 16S rRNA gene amplification, and Illumina sequencing

The DNA of the air samples was extracted from the Sterivex filters using a modified version of the protocol from Lever and al 2015 ((Lever et al. 2015), Supplementary Material – Protocol). A sterile Sterivex filter was used as a negative control. The rock samples were crushed into powder aseptically using a homemade tool designed for this purpose (Supplementary Material – Figure 1) The DNA of the different parts of the rocks and the soil samples were extracted using the DNeasy PowerMax Soil Kit (Qiagen, Hilden, Germany). DNA concentrations were checked for all samples using Qubit for high-sensitivity dsDNA (Thermo Fisher Scientific, Waltham, MA, USA).

The 16S rRNA gene sequencing method was used to effectively characterize the taxonomic composition of microbial communities, which is a primary focus of this study. The V4 region of the bacterial and archaeal 16S rRNA genes were amplified in triplicates from each DNA using the 515F (5'-GTGCCAGCMGCCGCGGTAA-3') and 806R (5'-GGACTACHVHHHTWTCTAAT-3') primers (Skirnisdottir et al. 2000) and the Q5 DNA polymerase (New England Biolabs, Ipswich, USA). Amplification was carried out according to Daussin et al. (2023b) with 2 ng/µl of DNA template for the rocks and soil, or 2–5 µl of the raw DNA solution extracted from the air samples. The master mix received an addition of bovine serum albumin (BSA) at a concentration of 1 µg/µl to facilitate amplification of the challenging samples. Thermal cycling conditions consisted of 98°C for 30 s; 30 cycles of 98°C for 10 s, 52°C for 30 s, and 72°C for 30 s; and 72°C for 2 min. Triplicate reactions were pooled, and amplicons were size selected from 1% (w/v) agarose gels using the Monarch DNA Gel Extraction Kit (New England Biolabs). The preparation of sequencing libraries and sequencing was done according to Bergsten et al. (2021).

### Comparison of the ASVs recovered with amplicon sequencing and cultivation

To compare the ASVs recovered from both cultivation (Daussin et al. 2023b) and amplicon sequencing, only the samples utilized in both methods were considered (Daussin et al. 2023b). A megablast was performed between the ASVs obtained with both methods before any decontamination process. Similar to the Daussin et al. (2023b) study, only matches with a minimum identity of 98.65% were retained.

### Data analysis

RStudio was used for bioinformatics and statistical analyses, employing R (v 3.6.0) and according to the DADA2 pipeline tutorial (1.8) (Callahan et al. 2016) for the lava rock and the soil samples, and the air samples separately. The two resulting Phyloseq objects were merged after the respective decontamination steps. The subsequent trimming criteria were applied: trimLeft = c(0, 0), maxN=0, maxEE=c(3, 3), and truncQ=2, and truncLen=c(240, 200) for the lava rock and the soil samples and truncLen=c(240, 220) for the air samples. Taxonomic assignments and contaminant identification were performed according to Bergsten et al. (2021) with the Decontam (v 1.6.0) package. The total number of reads per sample in the merged Phyloseq was equalized to a rarefaction depth corresponding to 90% of the minimum sample depth in the dataset, which was 5602 reads. All analyses were performed on the merged phyloseq object, without the controls, and all coding lines used on the phyloseq objects on R can be found in the Supplementary Material – Codes.

### Statistical analysis

Alpha diversity analysis was conducted in R using the Vegan package [17]. To assess the influence of different factors and confirm the alpha diversity results, an analysis of variance (ANOVA) was applied to the Shannon Indexes.

For beta diversity evaluation, data normalization was performed using the “rarefy_even_depth” method before conducting a Bray−Curtis-based nonmetric multidimensional scaling (NMDS). The statistical significance of factors such as the sampling site and year was determined using permutational multivariate analysis of variance (PERMANOVA).

Before executing the PERMANOVA analysis, we ensured the prerequisites were met by conducting preassessments using PERMDISP and Tukey’s honest significant differences tests. These evaluations confirmed significant statistical disparities in both dispersion between groups and groups means, thereby supporting the validity of our findings (Supplementary material – Table 2).

### Source prediction for the 1-year-old lava rock microbes in Surtsey

The Venn diagram created in Excel shows the common ASVs identified through the microbiome package (Lahti and Shetty 2019) among the air, the 1-year-old lava rock, and the soil of Surtsey. The SourceTracker package (Knights et al. 2011), utilizing Bayesian methods (McGhee et al. 2020), assessed the contributions of air and soil to the lava rocks, with each rock individually analyzed, considering air and soil from the same location as potential sources. To quantify the hypothetical contribution of each source to the microbial diversity of the 1-year-old lava rock, we performed a supplementary investigation in which we compared the presence of genera across the lava rocks and different environments (soil, air, fumarole, drill core, and seawater), suggesting potential dispersal between them. The speculative nature of this source prediction method arises from the occurrence of certain genera in multiple environments.

## Results

### Overview of the microbial diversity in the air and lava rock samples recovered from Surtsey and Fimmvörðuháls

**Figure 3.**
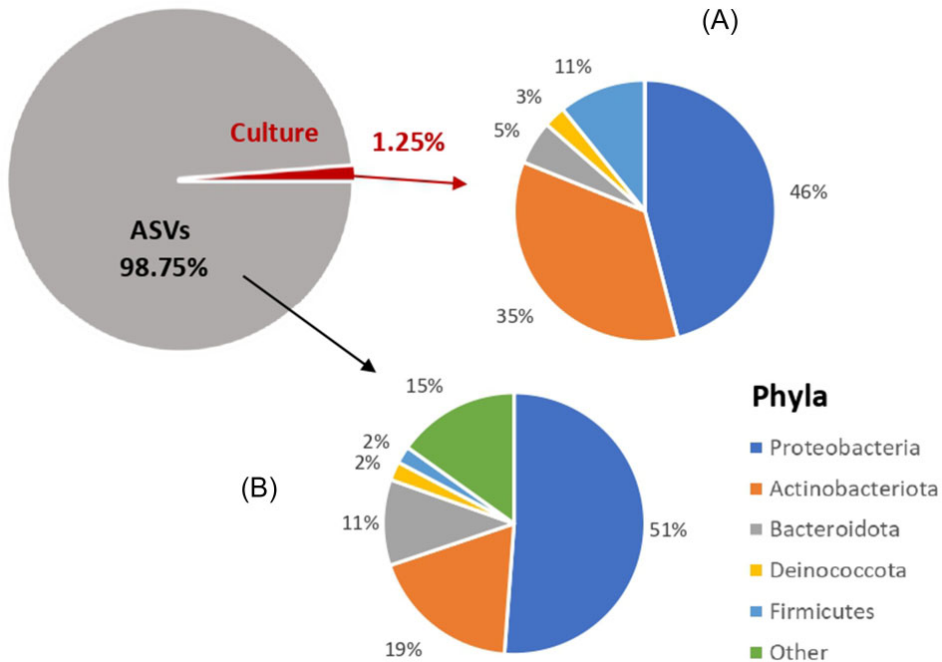
AVS as Phyla proportion of the prokaryotic cells obtained using culture-dependent (A) (Daussin et al. 2023a) and culture-independent (B) (this study) methods. A total of 1.25% of the ASVs obtained by amplicon sequencing were also detected using cultivation and 16S rRNA sequencing. The five major phyla detected by cultivation were also found by amplicon sequencing or 85% of the total ASVs. Proteobacteria and Actinobacteria were the dominant phyla, 97% and 54%, respectively, using both methods.

A total of 48 581 ASVs (archaea: 447, bacteria: 47 999, and NA: 135) were recovered from the 179 samples analyzed (Supplementary Material – Table 3). The rarefaction curves showed that all the libraries reached a point of saturation, indicating that the sequencing generated sufficient reads to capture the complete diversity of the samples (Supplementary Material – Figure 2). After performing a megablast, 174 ASVs representing the cultivable bacterial community obtained in a previous study (Daussin et al. 2023b) matched the amplicon results, corresponding to 1.25% of the total ASVs in these samples (Fig. 3).

Statistical analysis of the separate rock sections revealed only a minor difference in microbial communities between the top and the bottom of the rocks (Table 1). To enhance clarity and align with our goals, the microbial communities from all three sections will be combined and analyzed collectively.

**Table 1.** Statistical analysis of the microbial diversity. Statistically different parameters are underlined.

		Alpha diversity				Beta diversity	
		Observed		Shannon		PERMANOVA	
		*F* value	Pr(>F)	*F* value	Pr(>F)	*F* value	Pr(>F)
Air	Year	0.90	0.35	0.004	0.95	0.007	0.915
	BSA	0.13	0.72	0.28	0.60	0.7782	0.358
	Site	1.10	0.30	3.38	0.07	6.1845	0.01**
	Station	2.77	0.02*	6.37	7.81e–05***	5.27	0.001***
	Impinger	0.16	0.70	0.04	0.842	0.5428	0.441
1-year-old lava rocks	Year	NA	NA	NA	NA	NA	NA
	BSA	1.93	0.17	1.82	0.18	1.78	0.193
	Site	7.48	0.007**	5.98	0.016*	34.45	0.001***
	Station	3.45	0.002**	2.49	0.022*	8.02	0.001***
	Part	0.217	0.806	1.0	0.37	1.80	0.17
Lava rocks of Fimmvörðuháls	1-year-old vs 9-year-old	NA	NA	NA	NA	3.65	0.6
1-year-old lava rocks of Surtsey	Top-center	NA	NA	NA	NA	1.62	0.213
	Center-bottom	NA	NA	NA	NA	2.6	0.125
	Top–bottom	NA	NA	NA	NA	7.02	0.012*
	Type	NA	NA	NA	NA	3.7	0.024

*: Significant; **: Very Significant; ***: Highly Significant

**Figure 4.**
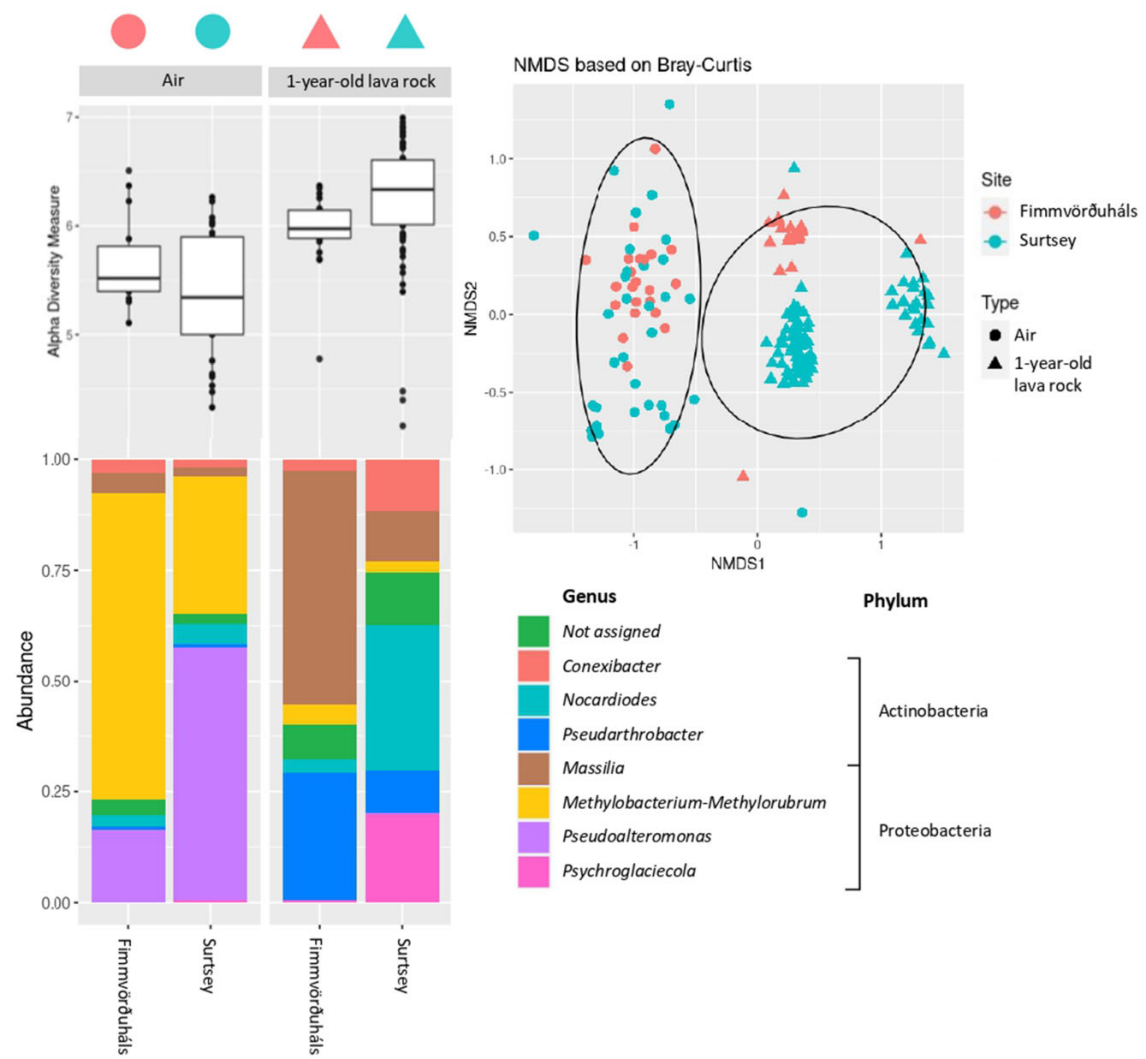
Alpha diversity, most abundant genera, and Bray–Curtis-based NMDS plot of the different samples recovered in this study (stress value = 0.139091). Circles in the NMDS plot are statistical ellipses for the different types of samples, the left one represents the air samples whereas the right circle represents the 1-year-old lava rock samples. The figure illustrates that the microbial diversity in the air samples from both sites is similar, while the communities inhabiting the 1-year-old lava rocks differ between the two sites.

The Bray–Curtis-based NMDS exhibited a stress value of 0.14, indicating the need for caution in interpreting the results. However, notable trends are clearly apparent and showed distinct microbial communities between the air and the 1-year-old lava rock samples (Fig. 4). However, microbial communities of the air samples recovered from Surtsey and Fimmvörðuháls were similar to each other whereas the 1-year-old lava rock communities exhibited differences between Surstey and Fimmvörðuháls. Furthermore, at Fimmvörðuháls, the microbial communities recovered from the 1-year-old lava rocks were similar to the communities on the 9-year-old lava rocks. The ANOVA for Shannon’s index showed significant differences between the microbial communities at the different sites, for both the air and the 1-year-old lava rocks. No significant differences were found between the communities recovered from the different sampling years, and with or without using BSA (Table 1). The alpha diversity was higher in the 1-year-old lava rock samples than in the air samples, at both sites. Moreover, the highest diversity was found in the rocks of Surtsey whereas the lowest diversity was found in the air of the same site (Fig. 4). The alpha diversity was similar for the 1-year-old and the 9-year-old lava rocks of Fimmvörðuháls.

### Community composition in the different samples of Surtsey and Fimmvörðuháls

In the air samples, cells affiliated with Proteobacteria represented the majority of the community (Fig. 4). The most abundant genera were similar for the two sites with a majority of *Pseudoalteromonas* and *Methylobacterium–Methylorubrum*, followed by *Nocardioides, Massilia*, and *Conexibacter*. The air of Fimmvörðuháls was dominated by *Methylobacterium–Methylorubrum* whereas the air of Surtsey was dominated by either *Pseudoalteromonas* or *Methylobacterium–Methylorubrum*, depending on the sampling station (Fig. 4). Representatives of the *Methylobacterium–Methylorubrum* genus were found in majority in the air above the Mávavarp, Tangi, and Viti stations, whereas representatives of the *Pseudoalteromonas* genus dominated the air above the Austur, Borhola, and Pálsbær stations (Supplementary Material – Figure 3).

In the 1-year-old lava rock samples, the majority of sequences were affiliated with Proteobacteria and Actinobacteria (Fig. 4), and clear differences were observed between the two sites. The 1-year-old lava rocks of Fimmvörðuháls were dominated by *Massilia* and *Pseudarthrobacter*, followed by *Methylobacterium–Methylorubrum*, whereas the l-year-old lava rocks of Surtsey were dominated by *Nocardiodes* and *Psychroglaciecola*, followed by *Conexibacter* and *Massilia* (Fig. 4). No difference was noticed between stations on each site (Supplementary Material – Figure 3).

**Figure 5.**
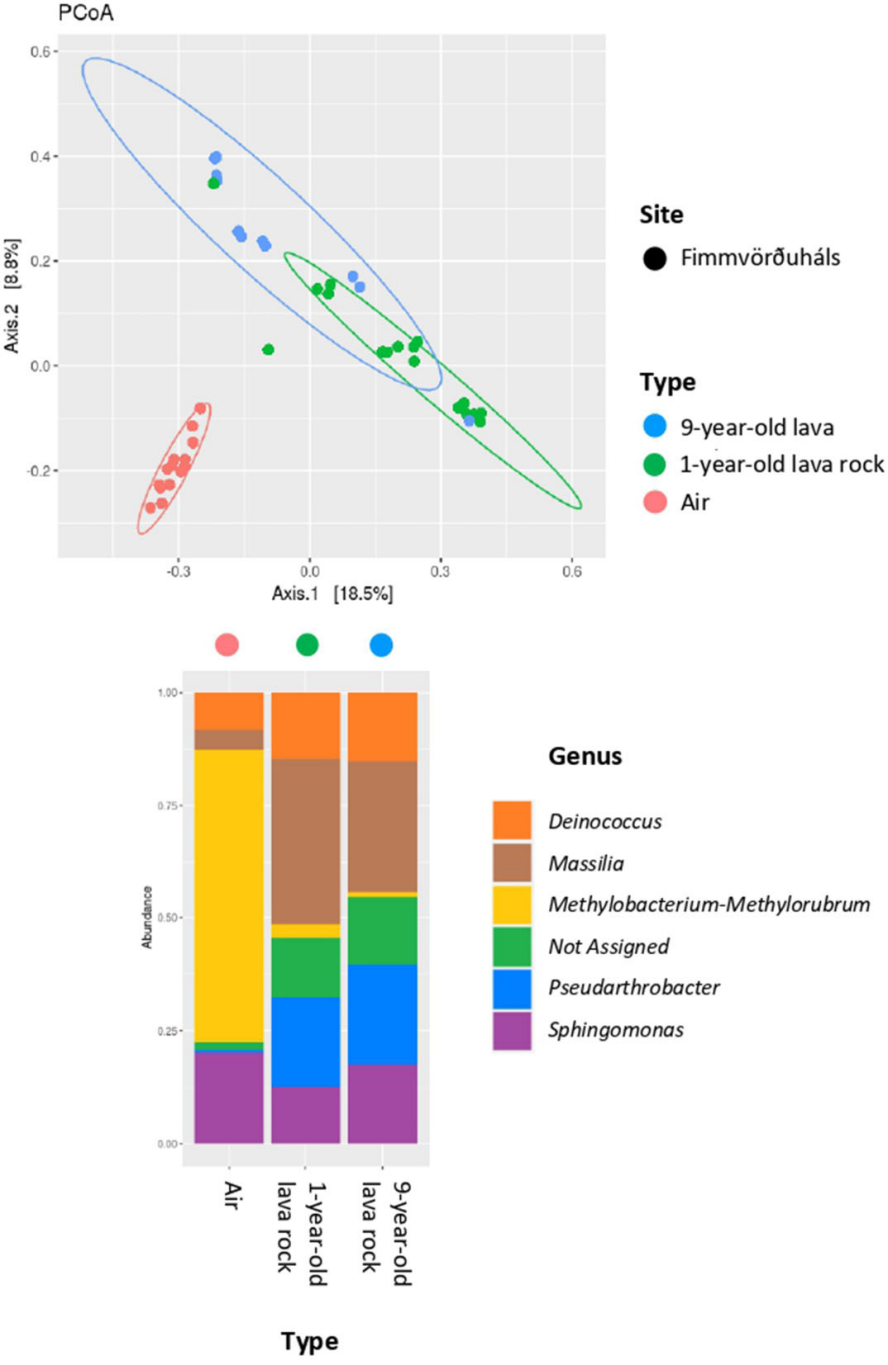
PCoA analysis and most abundant genera found in the different sample types recovered from Fimmvörðuháls. The microbial communities in the lava rocks exhibit a similar diversity both after 1 year and 9 years of exposure. However, these lava rock communities differ significantly from the microbial communities found in the air samples collected on the same site.

The microbial communities recovered from the 9-year-old lava rock samples of Fimmvörðuháls were dominated by representatives of the *Massilia* and *Pseudarthrobacter* genera (Fig. 5).

### Hypothetical prediction of the microbial origin in 1-year-old lava rock deposit at Surtsey

**Figure 6.**
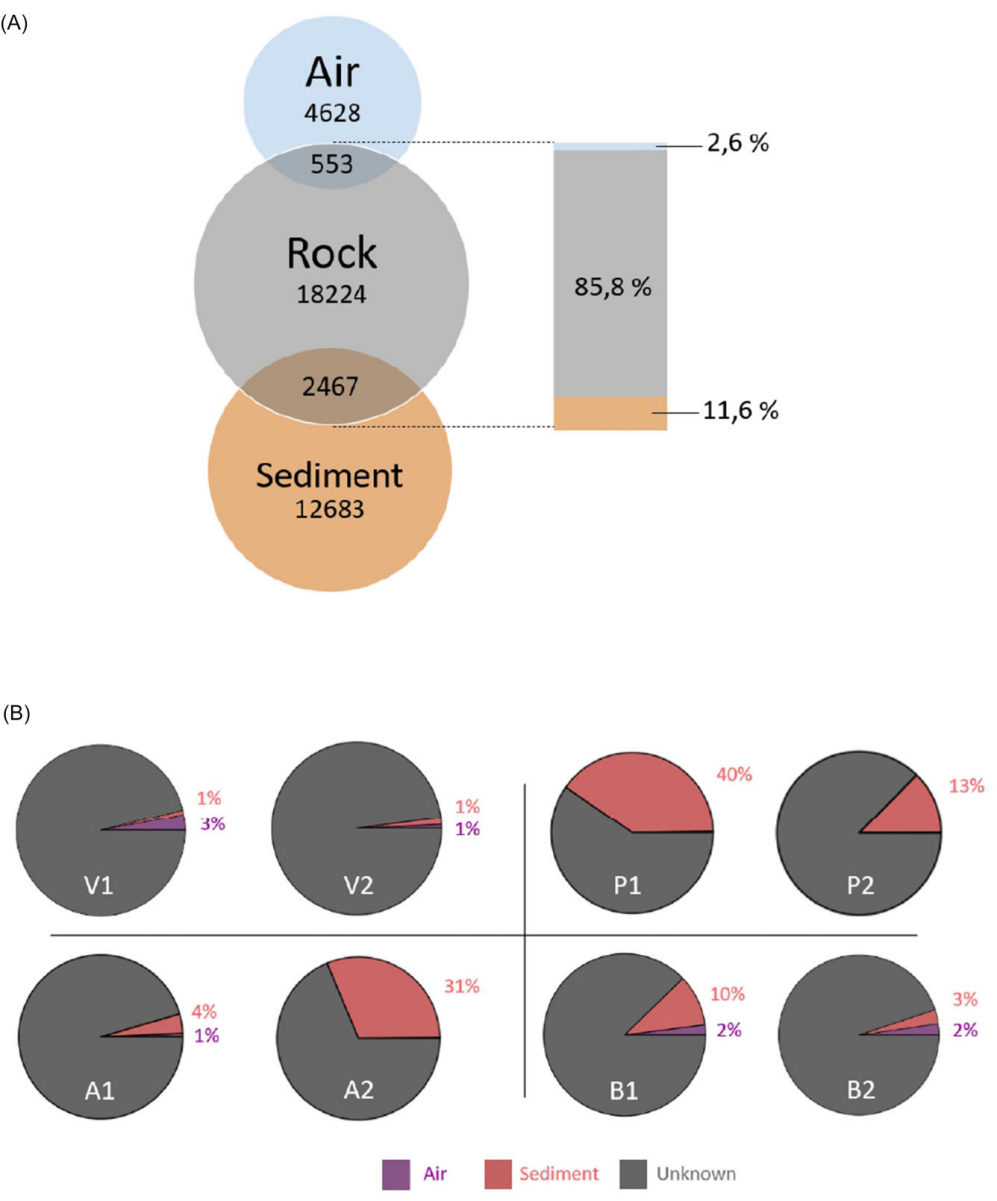
Venn diagram of the ASVs recovered from the different sample types in Surtsey (A, numbers represent ASVs) and SourceTracker result for 1-year-old lava rocks recovered from Surtsey (B) (each circle represents one rock, and the different sources are indicated with colors. V: Viti; P: Pálsbær; A: Austur; and B: Borhola).

The Venn diagrams of the different 1-year-old lava rocks recovered from Surtsey along with the corresponding air and soil samples showed that on average, 12.6% of the ASVs found in the 1-year-old lava rocks samples were also found in the underlying soil samples whereas only 2.6% of these ASVs were also found in the air samples (Fig. 6A). The SourceTracker analysis confirms this result with on average 12.9% of the microbes recovered from the 1-year-old lava rock in Surtsey coming from the underlying soil and 1.8% from the air (Fig. 6B). However, different proportions were observed in the different investigated sampling stations with a maximum of 40% of the microbes in the 1-year-old lava rock originating from the underlying soil at Pálsbær, and a minimum of 1% at Viti.

**Figure 7.**
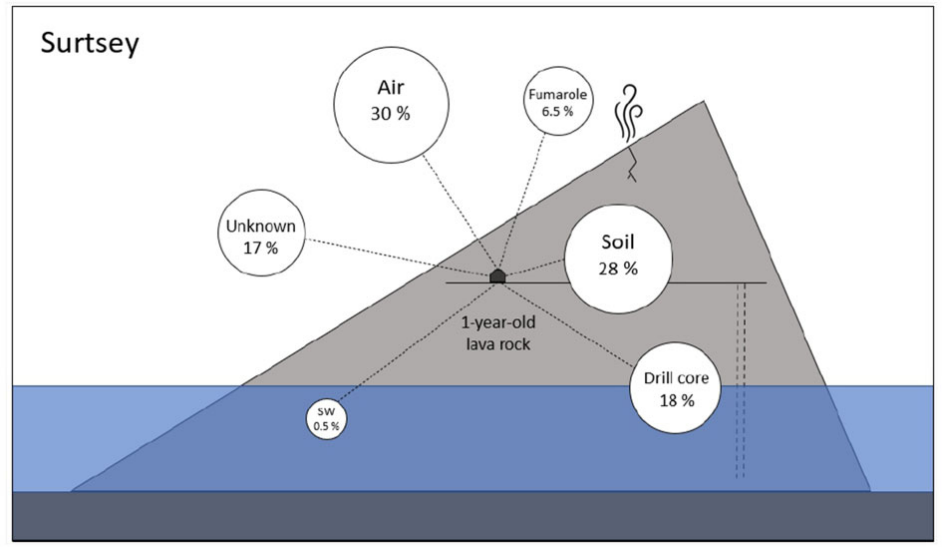
Schematic view of the potential sources and their relative contribution to the microbial diversity found in the 1-year-old lava rock of Surtsey. Numbers indicate the proportion of shared genera between the 1-year-old lava rocks and the different potential sources. (Sequences from drill core; SW: seawater and fumarole were acquired from Bergsten et al. 2021).

Additionally, 30% of the genera in the lava rocks after 1 year of exposure at Surtsey were also found in the air, and 28% in the soil (Fig. 7). Moreover, the contribution of the fumarole, drill core, and seawater samples led up to 25% of the total genera in the lava rock, leaving 17% of unknown-sourced genera. No correlation was observed between the drill core depth and the common genera with the 1-year-old lava rock at the surface (Supplementary Material – Figure 5). The 10 most abundant genera found in the lava rock were also the most abundant in the other potential sources, except for the seawater samples that only contained *Pseudoalteromonas* and *Alteromonas*. No *Psychroglaciecola* was found in common between the lava rocks and the drill core samples (Supplementary Material – Figure 4).

## Discussion

The air samples, which were taken during two consecutive summers, were characterized by a similar alpha and beta diversity regardless of the sampling year (Table 1). As demonstrated in a previous study (Daussin et al. 2023b), the air masses were coming roughly from the same direction in both years and at all sites. Based on the NMDS analysis (Fig. 4) of the atmospheric microbial communities, we conclude that these communities were relatively similar, even though there were significant differences identified by the ANOVA analysis (Table 1). These variations could be attributed to the presence of outliers, which are apparent on the NMDS plot, and to the dissimilar microbial compositions between the stations on Surtsey. Specifically, Pálbær, Austur, and Borhola exhibit the dominance of *Pseudoalteromonas*, while Mávavarp, Viti, and Tangi are characterized by a predominance of *Methylobacterium–Methylorubrum*.

The overall similarities between the different sites can be attributed to the fact that (i) the local surroundings and therefore sources of airborne cells at both sites share common traits, (ii) the backward trajectories indicate that the same areas contributed to the aerosols engaged in the long-distance dispersal, and (iii) the samples were collected during the summer period. Based on the similarity observed between the bacterial communities in aerosol samples collected during two consequent summers, we conclude that despite the relatively low temporal coverage, we could describe the taxonomy of depositing airborne cells during the summer periods well.

In the 1-year-old lava rock samples, the beta diversity analysis showed that the microbial communities differed between the sampling sites, which can be related to the differences in rock composition between the two sites. Lava rocks at Fimmvörðuháls contained approximately eight times more Fe_2_O_3_ than the lava rocks of Surtsey (Table 2). Moreover, it is essential to consider other potential contributors to this diversity and dynamics. Elements like physical structure and broader environmental and meteorological conditions, including spatial heterogeneity, external rain precipitation, and wind patterns, could also play significant roles in shaping these differences (Byloos et al. 2018). Collecting these additional data in a further study would be crucial for a more comprehensive understanding of the factors influencing microbial dynamics and diversity.

**Table 2.** Composition of the lava rocks of Surtsey and Fimmvörðuháls (Kelly et al. 2014, Schipper et al. 2015).

Site	Surtsey	Fimmvörðuháls
**Date**	19 May 1967	July–August 2010
**From**	Lava	Lava
**SiO_2_**	47.06	47.37
**TiO_2_**	2.04	2.90
**Al_2_O_**3**_**	13.61	14.88
**Fe_2_O_3_**	1.76	13.29
**FeO**	10.23	NA
**MnO**	0.19	0.19
**MgO**	11.82	8.11
**CaO**	10.62	9.50
**Na_2_O**	2.45	2.83
**K_2_O**	0.35	0.72
**P_2_O_5_**	0.19	0.39
**LOI**	0.21	NA
**Total**	100.53	99.71

While both air and lava rock environments host dominant genera with rapid adaptation capabilities, it is evident from the NMDS analysis and the culture-dependent study conducted on the same samples (Daussin et al. 2023b) that there are substantial differences between the microbial communities in these environments. Although the survival and initial establishment of cells on volcanic rocks may rely on similar adaptation properties as the ones needed for survival in the atmosphere, subsequent growth and success on lava rocks demand different characteristics. This disparity arises from both specific properties exhibited by the rock-dwelling microbes and the fundamental differences between the atmosphere and the rock environment itself. Unlike the atmosphere, lava rocks provide a wide range of microenvironments for microbial colonization, with cracks and fissures offering microorganisms several advantages, including protection from harsh environmental conditions such as extreme temperatures and UV radiation, water retention in pore spaces, limited competition for resources, limited predation from macroscopic organisms, and a stable physical structure for long-term colonization and adaptation (Antony et al. 2012). Moreover, the air samples represent the microbes found at the instant of the sampling, whereas the rock microbes reflect a longer colonization period. Additionally, it is important to note that the air samples represent the microbial composition at the time of sampling, while the rock samples reflect a longer period of colonization. These inherent distinctions may account for the observation that alpha diversity was highest in the rocks of Surtsey but lowest in the air at the same site.

We observed the transfer of bacterial cells between the soil and 1-year-old lava rocks, with four times more lava rock ASVs originating from soils compared to lava rock ASVs originating from the air. This could be attributed to the direct physical contact between rock and soils, which facilitates the exchange of microbial cells but could also be attributed to indirect contributions of soil ASVs via atmospheric dispersal. The latter is in line with studies that found that soils are the major source of atmospheric bacterial communities (Šantl-Temkiv et al. 2018, Archer et al. 2023). Moreover, our SourceTracker analysis highlighted spatial variations in microbial contributions from the soil across different sampling stations, indicating a site-dependent relationship. In Pálsbær and Austur, we observed a higher number of shared ASVs between the soil and the lava rocks. This can be attributed to the presence of similar volcanic rocks covering the soil in these areas, which is a result of their proximity to the respective west and east craters.

However, despite the significant exchange between soil and rock, most ASVs inhabiting the 1-year-old lava rocks came from unknown sources, distinct from both air and soil. This observation led us to hypothesize that these microbes likely settled over an extended period, originating from a diverse range of sources, contributing to the unique rock composition.

Moreover, we discovered that a significant proportion (83%) of the genera identified on the 1-year-old lava rocks at Surtsey were also found in the other investigated environments, suggesting potential exchange dynamics between them. Notably, an average of 71% of the genera present in the 1-year-old lava rock and other investigated sources were also detected in the air samples (as shown in Supplementary Material – Figure 6).

Additionally, the most abundant genera found in common between the 1-year-old lava rocks and the potential sources were *Nocardioides, Blastocatella, Hymenobacter, Spirosomona*, and *Deinococcus* (Supplementary Material – Figure 4). Most of them possess properties that might have helped them colonize a wide range of environments and/or survive atmospheric transportation (Daussin et al. 2023a). Indeed, representatives of the genus *Nocardiodes* have the capability to use various carbon and nitrogen sources (Gesheva and Vasileva-Tonkova 2012) *Hymenobacter* has previously been isolated from Icelandic environments and is a cold- and radiation-tolerant genus (Hilmisson 2020). Some *Spirosoma* species are also radiation-resistant (Park et al. 2021) and *Deinococcus* is well-known for its multiresistance capacity (Hirsch et al. 2004).

These observations suggest that the atmosphere could potentially serve as a vehicle for the transportation of a diverse range of stress-resistant microbial taxa from distant sources over longer periods of time. Furthermore, the significant exchange observed between soil and rock, alongside the shared genera between lava rocks and other environments, including air samples, indicates that habitat selection may be occurring from the air populations of microbes. To determine whether this is indeed the case, a long-term sampling strategy for atmospheric communities should be employed that would allow for a better understanding of its temporal heterogeneity.

Similar microbial communities were found after 1 year of exposure and after 9 years of exposure in Fimmvörðuháls with representatives of the genera *Massilia* and *Pseudarthrobacter* dominating (Fig. 5). In line with our findings, an earlier study also concluded that after a certain duration of exposure, the age of Icelandic basaltic rock does not have a significant influence on microbial communities (Byloos et al. 2018). One explanation could be that these communities were already well-established after 1 year, making it difficult for new depositing cells to colonize the rocks. It is also possible that the properties that helped these communities survive atmospheric stressors allowed them to settle and thrive sustainably on the rocks, thus facilitating their preferential colonization even after 9 years. For instance, certain *Massilia* species are known for possessing multiresistance properties that allow them to withstand various stresses present in both the air and rock environments (Sedláček et al. 2022). It is important to recognize the limitations of our method, especially the existence of certain genera in multiple environments. However, it allows us to capture broad trends among phylogenetically related groups that frequently share similar properties.

One limitation of this study is that the origin of the microbes in the 1-year-old lava rocks was only investigated at one sampling site. While the results provide valuable information about microbial communities at that location, it is possible that the results would differ if the analysis was applied to other sites. Another limitation is that the different potential sources were not sampled at the same time. To establish the link between the atmospheric and volcanic communities, as well as predict the sources of the microorganisms, microorganisms from the different environments would need to be sampled continuously over a long time while simultaneously the succession on volcanic rocks would be followed. Unfortunately, this is technically extremely challenging in the remote locations that we targeted. While acknowledging the limitations of the 16S rRNA sequencing, we opted for it due to its practicality for our research goals. We are aware of potential bias introduced by primer selection, as it can influence preferential amplification of certain microbial groups and result in taxonomic disparities. Additionally, our study did not measure the preferential settling of certain taxa through dry and wet processes, which could have influenced the microbial communities’ composition and distribution.

## Conclusion

In this study, we examined and compared the microbial communities from two different active volcanic sites in Iceland: Surtsey and Fimmvörðuháls. Airborne microbial communities were similar between sites while significant differences were found between the communities established on the lava rocks after 1-year exposure. Microbial communities did not change significantly between 1 and 9 years of exposure at Fimmvörðuháls, which implies that the bacterial communities are already well-established early in the process. Based on a comprehensive examination of bacterial communities that developed after 1 year of exposure on the lava rocks of Surtsey, we hypothesized that a significant portion of these communities could have potentially originated from local surrounding sources and been dispersed to the rocks through the atmosphere over time.

Overall, we conclude that atmospheric dispersal is a key factor involved in the colonization of volcanic rocks, and the cells that settle in these newly formed environments are responsible for establishing unique and diverse communities in less than 1 year.

Understanding the link between atmospheric and lithospheric microbial communities has multifaceted implications. It aids in assessing the resilience of microbial communities in response to environmental changes and disturbances. Additionally, it advances our scientific comprehension of microbial ecology in extreme environments, with potential implications for fields like geomicrobiology, astrobiology, and biogeography.

## Code availability

The pipeline utilized for analyzing the dataset reported in this manuscript can be accessed at https://benjjneb.github.io/dada2/tutorial.html, and the code used to build figures can be found in Supplementary Material—Code.

## Supplementary Material

xtae016_Supplemental_Files

## Data Availability

The data sets (accession number PRJEB64516) for this study can be found in the European Nucleotide Archive at EMBL-EBI (https://www.ebi.ac.uk/ena/browser/view/PRJEB64516).
