# Supplementary material for: Atmospheric dispersal shapes rapid bacterial colonization of Icelandic Lava Rocks
Source: FEMS Microbes. 2024 May 24;5:xtae016. doi: 10.1093/femsmc/xtae016 (PMC11173176; doi:10.1093/femsmc/xtae016)
Supplement: xtae016_Supplemental_Files [file xtae016_supplemental_files.zip › Supplementary Material 240321.docx]

**Supplementary Material**

**Supplementary Material - Protocol 1.** DNA extraction protocol from a sterivex filter (modified protocol from Lever et al., 2015)

To extract DNA from Sterivex filters in a sterile manner, the filter was removed and cut into small pieces with a sterile scalpel and transferred to a 2 mL-Eppendorf tube. The filter was entirely soaked in 200 µL of 10 mM dNTPs, followed by the addition of 1 mL of Lysis solution, and mixing by inversion. The mixture was frozen at -80 °C for a minimum of 40 minutes and thawed, followed by bead-beating with 0.1mm silica beads for 10 seconds and 1 minute on ice, repeated three times. The liquid was then transferred to a cold 15 mL-Falcon tube and 1 mL of Phenol:Chloroform:Isoamyl Alcohol (25:24:1, v/v) (PCI) was added under a chloroform-ready hood, mixed by inversion, and centrifuged at 4 °C for 18 minutes at 5,500 x g. The supernatant was transferred to a clean cold Falcon tube and 1 mL of PCI was added, followed by repeating the centrifugation step. The supernatant was carefully transferred to a clean cold 2 mL-Eppendorf tube, and 16 µL of Polyethylene Glycol (PEG 400) solution was added and mixed by inversion, followed by the addition of 80 µL of 5 M NaCl and 1.2 mL of isopropanol. The mixture was incubated in the dark at -22 °C for a minimum of 2 hours, followed by centrifugation at 4 °C and 16,000 x g for 25 minutes. The supernatant was removed, and the pellet was resuspended with 500 µL of 70% EtOH, centrifuged at 4 °C and 16,000 x g for 5 minutes, and the supernatant was removed. The pellet was air-dried, and then dissolved in 100 µL of sterile dH2O on ice.

The lysis solution used in this experiment contained Tris HCl at a concentration of 30 mM, EDTA at a concentration of 30 mM, Guanadium hydrochloride at a concentration of 800 mM, and Triton X-100 at a concentration of 0.5%.


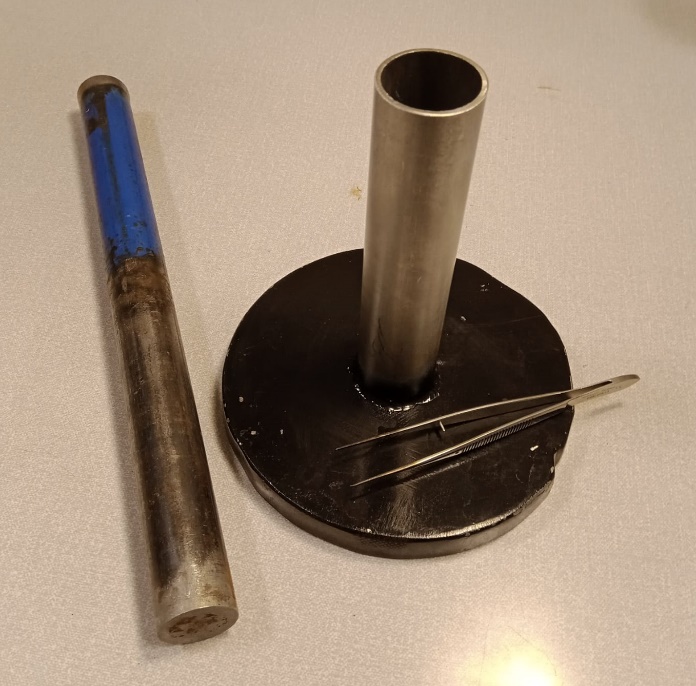


**Supplementary Material - Figure 1.** Picture of the homemade tool used to crush pieces of rocks into powder.


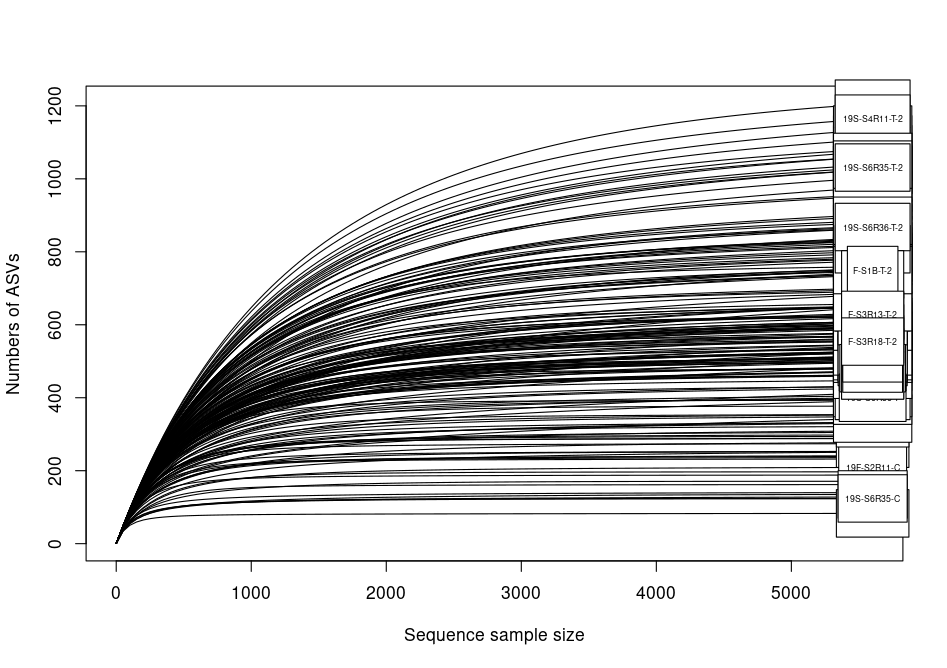


**Supplementary Material - Figure 2.** Rarefaction curves of the merged samples after subsampling to 5,062 reads per sample.


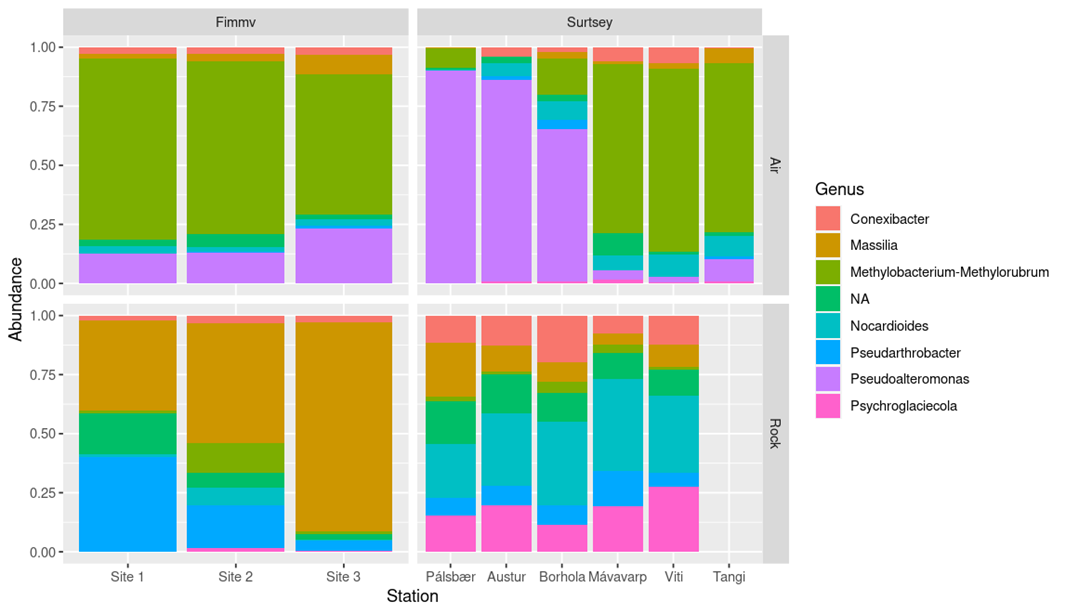


**Supplementary Material - Figure 3**. Most abundant genera found in the air and in the 1-year-old lava rock samples recovered from the different stations of Fimmvörðuháls and Surtsey.

**Supplementary Material - Figure 4.** 100 most abundant common genera found in the 1-year-old lava rocks of Surtsey and the investigated sources. The number 1 indicates presence whereas 0 indicates absence.


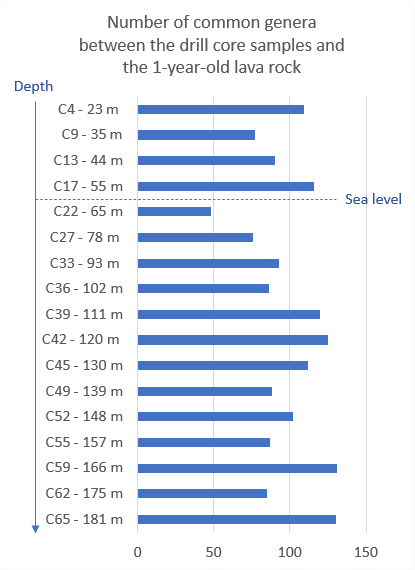


**Supplementary Material - Figure 5.** Number of genera found in the 1-year-old lava rocks of Surtsey and in each drill core sample.


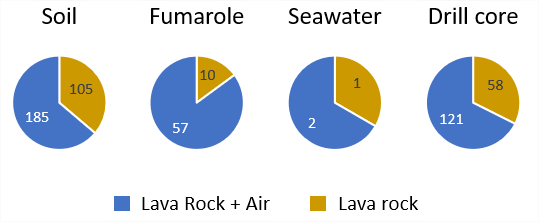


**Supplementary Material - Figure 6.** The number of genera found in common between the investigated sources, the lava rocks, and the air.

**Supplementary Material - Table 1**. Characteristics of the sampling stations and the number of samples. Eyja.: Eyjafjallajökull.- means zero.

| Location | Station - Abbreviation | Coordinates | Elevation | Characteristics | Year | Air samples | Rock samples | | Sediment samples |
| --- | --- | --- | --- | --- | --- | --- | --- | --- | --- |
|  |  |  |  |  |  |  | 1-year-old | 9-year-old |  |
| Surtsey | Mávavarp - M | 63°17.880‘N  20°36.092´W | 50 m | Gull nesting area - vegetation | 2018 | 2 | - | - | - |
|  |  |  |  |  | 2019 | 2 | 2 | - | 2 |
|  | Borhola - B | 63°18.097´N  20°36.010´W | 66 m | Boreholes – Human activity | 2018 | 2 | - | - | - |
|  |  |  |  |  | 2019 | 2 | 2 | - | 2 |
|  | Viti - V | 63°18.244´N  20°36.062´W | 166 m | Lighthouse – Highest point | 2018 | 2 | - | - | - |
|  |  |  |  |  | 2019 | 2 | 2 | - | 2 |
|  | Tangi - T | 63°18.455´N  20°36.011´W | 11 m | Peninsula – Rocks | 2018 | 2 | - | - | - |
|  |  |  |  |  | 2019 | 2 | - | - | - |
|  | Pálsbær - P | 63°18.047´N  20°35.828´W | 38 m | Human hut | 2018 | - | - | - | - |
|  |  |  |  |  | 2019 | 2 | 2 | - | 2 |
|  | Austur - A | 63°18.153´N  20°36.609´W | 108 m | Crater | 2018 | - | - | - | - |
|  |  |  |  |  | 2019 | - | 2 | - | 2 |
| Fimmvörðuháls | Top Eyja. – Site 1 | 63°37.985‘N  19°26.466´W | 1,036 m | Crater | 2018 | 2 | - | - | - |
|  |  |  |  |  | 2019 | - | 2 | 1 | - |
|  | Lava Eyja. – Site 2 | 63°38.182‘N  19°25.991´W | 1,030 m | Lava field | 2018 | 2 | - | - | - |
|  |  |  |  |  | 2019 | - | 2 | - | - |
|  | Hut - Site 3 | 63°36.654‘N  19°26.489´W | 875 m | Human hut | 2018 | 2 | - | - | - |
|  |  |  |  |  | 2019 | - | 2 | 1 | - |

**Supplementary Material - Table 2**. Summary of the statistical test results.

**Supplementary Material - Table 3.** Summary of the samples and ASVs obtained in this study. (18F: Fimmvörðuháls 2018; 18S: Surtsey 2018; 19F: Fimmvörðuháls 2019; 19S: Fimmvörðuháls 2019; Exp: Experiments).

|  | | **Phyloseq 1** | | **Phyloseq 2** | | |  | **Merged Phyloseq 1 and 2** |
| --- | --- | --- | --- | --- | --- | --- | --- | --- |
|  |  | **Air** | | **Rocks** | | **Sediments** | **Exp** | **Sum**  **(Without controls)** |
| **Sample**  **numbers** | **Sampling**  **acronym** | **samples** | **controls** | **1-year-old** | **9-years-old** | **samples** | **controls** | **179** |
|  | **18F** | 9 | 7 | 0 | 0 | 0 | 3 |  |
|  | **18S** | 7 | 1 | 0 | 0 | 0 |  |  |
|  | **19F** | 5 | 1 | 22 | 11 | 0 |  |  |
|  | **19S** | 18 | 8 | 81 | 0 | 26 |  |  |
|  | **Sum** | 39 | 17 | 103 | 11 | 26 | 3 |  |
| **ASV**  **numbers** | **Before Decontam** | 12,496 | | 38,788 | | | | 51284 |
|  | **After Decontam** | 11,586 | | 38,281 | | | | **48,581** |
| **Domain classification** | | | | | | | Archaea | 447 |
|  |  |  |  |  |  |  | Bacteria | 47,999 |
|  |  |  |  |  |  |  | Unassigned | 135 |

**Supplementary Material - Codes**

*psd is the phyloseq object*

**Alpha diversity (Figure 3, Table 1)**

plot_richness(psd, x="Site", measures=c("Shannon")) + geom_boxplot()+ facet_grid(. ~ Type)

alpha.diversity <- estimate_richness(psd, measures = c("Observed", "Shannon", "InvSimpson"))

metadatr <- cbind(sample_data(psd), alpha.diversity)

anova_result.o.ST <- aov(Observed ~ Station, metadatr)

anova_result.s.ST <- aov(Shannon ~ Station, metadatr)

summary(anova_result.o.ST)

summary(anova_result.s.ST)

**Beta diversity/NMDS (Figure 3, Figure 4)**

set.seed(1)

psdn = rarefy_even_depth(psd, rngseed=1, sample.size=0.9*min(sample_sums(psd)), replace=F)

ps.dord <- ordinate(psdn, "NMDS", distance = "bray")

plot_ordination(psdn, ps.dord, shape="Type",color="Site",title="NMDS based on Bray-Curtis") + geom_point(size=3) + stat_ellipse(aes(group=Type))

**Permanova analysis (Table 1)**

set.seed(1)

erie_bray <- phyloseq::distance(psdn, method = "bray")

sampledft <- data.frame(sample_data(psdn))

beta <- betadisper(erie_bray, sampledft$Type)

permutest(beta)

**Stacked bar plots (Figure 3, Figure 4)**

prune.dat <- prune_taxa(taxa_sums(psd) > 2, psd)

top20 <- names(sort(taxa_sums(psd), decreasing=TRUE)[1:20])

top20

dat.aglo = tax_glom(psd, taxrank = "Genus")

dat.trans = transform_sample_counts(dat.aglo, function(x) x/sum(x))

prune.dat.two = prune_taxa(top20, dat.trans)

dat.dataframe = psmelt(prune.dat.two)

dat.agr = aggregate(Abundance~Site+Type+Genus, data=dat.dataframe, FUN=mean)

ggplot(dat.agr, aes(x=Site, y=Abundance, fill=Genus)) + geom_bar(stat="identity",position="fill") + facet_grid(~Type, scale="free")
