# Supplementary material for: Atmospheric dispersal shapes rapid bacterial colonization of Icelandic Lava Rocks
Source: FEMS Microbes. 2024 May 24;5:xtae016. doi: 10.1093/femsmc/xtae016 (PMC11173176; doi:10.1093/femsmc/xtae016)
Supplement: xtae016_Supplemental_Files [file xtae016_supplemental_files.zip › FEMSMC-2023-045.R2 one sentence summary.docx]

Atmospheric dispersal plays a crucial role in colonizing Icelandic volcanic rocks, leading to the rapid formation of unique and diverse communities within just one year.
