# Supplementary material for: Atmospheric dispersal shapes rapid bacterial colonization of Icelandic Lava Rocks
Source: FEMS Microbes. 2024 May 24;5:xtae016. doi: 10.1093/femsmc/xtae016 (PMC11173176; doi:10.1093/femsmc/xtae016)
Supplement: xtae016_Supplemental_Files [file xtae016_supplemental_files.zip › FEMSMC-2023-045.r2 supplementary data - reviews and author response.docx]

**Reviews of manuscript FEMSMC-2023-045**

**Reviewer 1: Stephen Pointing, National University of Singapore**

**Recommendation: Minor revision**

The manuscript compared microbial diversity from atmospheric and lithospheric samples at volcanic locations in Iceland. The experimental work employed rRNA sequencing, culturing, and various analyses to identify the communities and potential sources from a large number of samples (n = 179). Each air sample was taken in duplicate but it is not clear how many replicates were taken for each soil location and how they were treated in the analyses, so as a minor suggestion for improvement this could be clarified in the text. The results showed that atmospheric samples were similar, but the lithosphere samples displayed greater variation linked to their age since exposure. Source tracking highlighted the importance of air and soil as a source for microbial immigration (with a very nice synthesis figure for this finding, Fig. 6). The manuscript is well written and appropriately referenced throughout. Overall, the findings advance our understanding of how volcanic landscapes are colonized by microorganisms.

**Reviewer 2: Rebecca Prescott**

**Recommendation: Major Revision**

This paper looks at microbial communities from very recent lava flows/rocks in 2 Icelandic

sites, and compares those communities to airborne microbial communities to address the

important question of what are the source microbial populations for new volcanic rock after an

eruption. This is a very interesting and important question, with implications for up-coming

moon and Mars missions, as well as studies of volcanic island nations and the breakdown of

lava rock into soils.

General Review:

Overall, the questions addressed and the methods used are very good, and I believe the paper

should be published, as this is a largely unknown area of research. The bioinformatic methods

are well explained and the authors used appropriate methods and analyses from what is

written. Figures are very good and readable. However, there are mentions of methods, data,

and analyses in the results and/or discussion that are not given in the methods, and this issue

needs to be addressed before publication. I have mentioned the items I noticed below in the

specific line reviews. For example, there is mention of a fumarole, drill core, and saltwater

sample in the discussion, that are not mentioned previously in the paper.

My biggest concern is the dependence on the PCoA analysis of this study, which needs to be

corrected and further explored (see below). I would also comment that the analysis of the rocks

composition should be in the paper, not supplemental. Without these data, its difficult to

explain the variation in the samples well, especially with the variation the iron.

There are many good ideas, and even literature review in the discussion that really would make

the paper stronger if there was some mention of them in the Introduction. In general, I think the

authors need to provide more examples/details in this manuscript, rather than simply listing a

reference for the reader. Same goes for the methods. I don’t know how the samples were

collected, what devices were used to collect the air samples, etc.

PCoA analysis (Figure 3): The percent variability captured on the x and y axis is quite low (less

than 10%), which suggests that this particular metric (Bray-Curtis) or method (PCoA) is not

doing a good job of representing the highly complex y-variable data in a 2-dimensional image

(something like 30% on PCO 1 suggests the image is representative of the higher dimensional

data). Therefore, the image is not meaningful. I would suggest that the authors try using

UniFrac metric instead in a PCoA, as it may capture more of the variability and would then be

worth including in Figure 3. Otherwise I would remove it from the manuscript as it currently is.

Unifrac is available in QIIME2 pipelines, or through EDGE Bioinformatics (which uses the

QIIME2 Pipeline). Another option is to use an NMDS rather than PCoA with Bray-Curtis, but

suspect you will see the same issue: a high stress value that will show that the graphic is not

an acceptable representation of the higher dimensional data. UniFrac usually does a better job

at capturing the variability in microbial datasets, so it would be worth trying. Since the authors

are relying on this analysis and ordination considerably in their conclusions, I believe this issue

needs to be addressed before publication, with at least exploration of other metrics and

methods to better assess the groupings of their sampling sites.

I am not clear on how the air samples (or other samples) were collected, and pictures of any air

collecting devices would help (same for instruments for the rocks, if any were used).

Specific Line review:

Line 118-119: did the authors use 16S rRNA sequencing to determine there were no

differences in the top, middle, bottom of the rocks? I would give a little bit more detail here.

Line 131: After Lever et al. - should there be a date of publication here?

Line 132: can a picture of the tool be provided in Supplemental?

Line 236: With the unidentified genera, can you provide a class or order level assessment?

Line 246: what is the SourceTracking analysis (its also listed as two words: Source Tracker -

decide which way is correct)? I am confused about what this actually is, and it probably needs

to be more clearly defined then, and the methods of that written out.

Line 257: where are the fumarole, Drill Core, seawater samples coming from? I believe this is

the first mention of them in the manuscript (my apologies if I missed it), but its not clear at the

moment while reading it.

Line 269: This is the first mention of the HYSPLIT model in the paper, and this is the

discussion. Please make sure to add the details of any methods used in the methods, that you

want to discuss in the the results or Discussion.

Line 289: The composition of the rocks is very important in this study, and should be moved

from the Supp. Materials to main body. Along with the methods of that work.

Line 292: were any of these data collected or looked up in meteorological databases/

information? If not, this could be very useful information that might help explain the variations

you see in samples.

Line 295: this is a very interesting comment. Can you connect better to your data? I don’t know

what groups you are referring to that have rapid adaptation capabilities? This is an area you

could expand on in the Introduction as well - something along the lines of life on rocks and a

brief review of that literature.

Line 335-336: what is the “genera-based source prediction”? I did a search and this is the only

place I can find this phrase. I think you mention looking at the genera-level data earlier in the

results, but is not clear if that is what you mean here? Or some other method?

**Editor Decision: Major Revision**

**Author's Response to Decision Letter for (FEMSMC-2023-045)**

Response to Reviewer 1:

Thank you for your review and comment. We have incorporated your suggestions to enhance the manuscript.

----------

Reviewer 1: Each air sample was taken in duplicate but it is not clear how many replicates were taken for each soil location and how they were treated in the analyses, so as a minor suggestion for improvement this could be clarified in the text.

Authors: We understand the reviewer's concern about the clarity of our sampling methods. Since we are using the same samples as in a previous study and to avoid redundancy and plagiarism, we referred to that publication. However, for better clarity, we added a short paragraph with a brief description of our sampling method and a picture of our air sampler:

Page 4, lines 117-122: We used air and lava rock samples previously collected as described in our prior publication [7]. Briefly, we employed High-Flow-Rate impingers (Figure 2) for air sample collection, and recovered on-site autoclaved lava rocks after one year, denoted as "1-year-old lava rocks." Additionally, non-autoclaved 9-year-old lava rocks from Fimmvörðuháls were designated as "9-years-old lava rocks".

Moreover, we have updated our summary table reference to include the number of recovered samples:

Page 5, lines 134-136: The coordinates, characteristics, number, and type of sample recovered from the different stations can be found in Supplementary Material - Table 1.

-----------------------------------------------------------------------------------------------------------------------------------------------------

Response to Reviewer 2:

Thank you for your valuable comments and the time you took to review our work. Your insights are greatly appreciated, and we have carefully considered and implemented your suggestions to improve the quality of the manuscript.

------------------

Reviewer 2: Line 118-119: did the authors use 16S rRNA sequencing to determine there were no differences in the top, middle, and bottom of the rocks? I would give a little bit more detail here.

Authors: Thank you for bringing this to our attention. Indeed, we used statistical analyses based on the 16S rRNA sequencing to determine the differences in the microbial communities of the different parts of the lava rocks. We added information in the “Material and Method” and “Results” parts for clarity:

In the Material and Method:

Page 5, lines 126-127: The 1-year-old lava rocks were fragmented using a sterilized hammer to facilitate analyses of the top, middle, and bottom parts.

Page 5, line 142: The DNA of the different parts of the rocks and the soil samples were extracted using the DNeasy PowerMax Soil Kit (Qiagen, Hilden, Germany)

In the Results:

Page 7, lines 212-215: Statistical analysis of the separate rock sections revealed only a minor difference in microbial communities between the top and the bottom of the rocks (Table 1). To enhance clarity and align with our goals, the microbial communities from all three sections will be combined and analyzed collectively.

------------------

Reviewer 2: Line 131: After Lever et al. - should there be a date of publication here?

Authors: Thank you for pointing out this mistake, it was corrected:

Page 5, line 139: The DNA of the air samples was extracted from the Sterivex filters using a modified version of the protocol from Lever and al 2015 ([10], Supplementary Material – Protocol).

------------------

Reviewer 2: Line 132: can a picture of the tool be provided in Supplemental?

Authors: It is now provided in Supplementary Material – Figure 1.

------------------

Reviewer 2: - For example, there is mention of a fumarole, drill core, and saltwater sample in the discussion, which are not mentioned previously in the paper.

Line 257: where are the fumarole, Drill Core, seawater samples coming from? I believe this is the first mention of them in the manuscript (my apologies if I missed it), but its not clear at the moment while reading it.

Authors: Thank you for these comments. We acknowledge that mentioning these samples earlier in the manuscript will make it clearer. A new sentence was added to the “site description and sample collection” part of the Material and Method:

Page 5, lines 130-132: Additionally, microbial sequences from fumarole, drill core, and seawater samples collected on Surtsey by Bergsten et al. (2021) [9] were incorporated into this study to explore potential sources for the 1-year-old lava rocks of Surtsey.

------------------

Reviewer 2: - I would also comment that the analysis of the rocks composition should be in the paper, not supplemental. Without these data, its difficult to explain the variation in the samples well, especially with the variation the iron.

Line 289: The composition of the rocks is very important in this study, and should be moved from the Supp. Materials to main body. Along with the methods of that work.

Authors: Based on your comment, we make is more apparent that the compositions were retrieved from the literature and move the Table we made from Supplementary Materials to the main body:

Page 10, lines 298-300: Lava rocks at Fimmvörðuháls contained approximately 8 times more Fe2O3 than the lava rocks of Surtsey (Table 2) (18).

------------------

Reviewer 2: - Same goes for the methods. I don’t know how the samples were collected, what devices were used to collect the air samples, etc.

- I am not clear on how the air samples (or other samples) were collected, and pictures of any air

collecting devices would help (same for instruments for the rocks, if any were used).

Authors: We understand the reviewer's concern about the clarity of our sampling methods. Since we are using the same samples as in a previous study and to avoid redundancy and plagiarism, we referred to that publication. However, for better clarity, we added a short paragraph with a brief description of our sampling method, and a picture of our air sampler:

Page 4, lines 117-122: We used air and lava rock samples previously collected as described in our prior publication [7]. Briefly, we employed High-Flow-Rate impingers (Figure 2) for air sample collection, and recovered on-site autoclaved lava rocks after one year, denoted as "1-year-old lava rocks." Additionally, non-autoclaved 9-year-old lava rocks from Fimmvörðuháls were designated as "9-years-old lava rocks". For detailed information on procedures, controls, and other aspects, please consult our previous publication.

------------------

Reviewer 2: - PCoA analysis (Figure 3): The percent variability captured on the x and y axis is quite low (less than 10%), which suggests that this particular metric (Bray-Curtis) or method (PCoA) is not doing a good job of representing the highly complex y-variable data in a 2-dimensional image (something like 30% on PCO 1 suggests the image is representative of the higher dimensional data).

Therefore, the image is not meaningful. I would suggest that the authors try using UniFrac metric instead in a PCoA, as it may capture more of the variability and would then be worth including in Figure 3. Otherwise I would remove it from the manuscript as it currently is. Unifrac is available in QIIME2 pipelines, or through EDGE Bioinformatics (which uses the QIIME2 Pipeline). Another option is to use an NMDS rather than PCoA with Bray-Curtis, but suspect you will see the same issue: a high stress value that will show that the graphic is not an acceptable representation of the higher dimensional data. UniFrac usually does a better job at capturing the variability in microbial datasets, so it would be worth trying. Since the authors are relying on this analysis and ordination considerably in their conclusions, I believe this issue needs to be addressed before publication, with at least exploration of other metrics and methods to better assess the groupings of their sampling sites.

Authors: Thank you for this relevant and constructive comment. The PCoA using the UniFrac metric revealed a comparable percentage of variability, with 10.3% on the x-axis and 5.5% on the y-axis. However, the NMDS analysis using UniFrac exhibited a stress value of 0.15, and the NMDS using the Bray-Curtis metric had a slightly lower stress value of 0.14, indicating a more effective representation of the higher dimensional data. Consequently, we have opted to present the NMDS plot using the Bray-Curtis metric in Figure 3, replacing the initial PCoA based on Bray-Curtis. References to the previous PCoA plot were modified in the text to the NMDS.

------------------

Reviewer 2: Line 236: With the unidentified genera, can you provide a class or order level assessment?

Authors: Our focus with this picture was on highlighting these similar diversity patterns rather than providing specific taxonomic classifications and we intentionally refrained from providing a class or order level assessment. To enhance clarity and prevent confusion, we've opted to remove any reference to these unidentified genera from the text.

------------------

Reviewer 2: Line 269: This is the first mention of the HYSPLIT model in the paper, and this is the discussion. Please make sure to add the details of any methods used in the methods, that you want to discuss in the results or Discussion.

Authors: Thank you for pointing this out. To avoid redundancy and plagiarism, we referenced our previous study where the details can be found.

Page 10, lines 279-281: As demonstrated in a previous study [7], the air masses were coming roughly from the same direction in both years and at all sites.

------------------

Reviewer 2: Line 292: were any of these data collected or looked up in meteorological databases/information? If not, this could be very useful information that might help explain the variations you see in samples.

Authors: Due to the remote locations of our sampling sites, we did not have the opportunity to collect or access meteorological data from databases during the current study. However, we acknowledge the significance of meteorological information in understanding variations observed in our samples and therefore modify the text to highlight its potential significance:

Page 10, lines 303-305: Collecting these additional data in a further study would be crucial for a more comprehensive understanding of the factors influencing microbial dynamics and diversity.

------------------

Reviewer 2: Line 295: this is a very interesting comment. Can you connect better to your data? I don’t know what groups you are referring to that have rapid adaptation capabilities? This is an area you could expand on in the Introduction as well - something along the lines of life on rocks and a brief review of that literature.

Authors: Thank you for your feedback. As the groups demonstrating rapid adaptation capabilities will be described later in the manuscript and to maintain clarity in this section, we deleted this part of the sentence.

Following your advice, we have included a few lines in the introduction about colonization:

Page 3, lines 70-73: The process of colonization is influenced by mineral properties, soil pH, and microbial traits. Notably, bacterial binding to minerals, facilitated by extracellular polysaccharides and proteins, is crucial for stable colonization [5], but is notably reduced in acidic and alkaline soils [6].

------------------

Reviewer 2: Line 246: what is the SourceTracking analysis (its also listed as two words: Source Tracker - decide which way is correct)? I am confused about what this actually is, and it probably needs to be more clearly defined then, and the methods of that written out.

Line 335-336: what is the “genera-based source prediction”? I did a search and this is the only place I can find this phrase. I think you mention looking at the genera-level data earlier in the results, but is not clear if that is what you mean here? Or some other method?

Authors: Thank you for bringing up this concern, addressing this confusion about the methods used to determine the potential sources of the microbes will enhance the overall clarity of the manuscript. The correct term is "SourceTracker," a program specifically designed to forecast the origins of microbial communities within a given set of input samples. To rectify this and better present the “genera-based source prediction”, we have modified a paragraph in the methods section:

Page 6, lines 187-200: Venn diagrams and SourceTracker analyses were used to determine the proportion of soil and air-originated microbes in the 1-year-old lava rocks of Surtsey. Additionally, we employed a method based on genera to explore the potential origin of microbes that didn't come from either air or soil.

The Venn diagram created in Excel shows the common ASVs identified through the microbiome package [13] among the air, the 1-year-old lava rock, and the soil of Surtsey. The SourceTracker package [14], utilizing Bayesian methods [15], assessed the contributions of air and soil to the lava rocks, with each rock individually analyzed, considering air and soil from the same location as potential sources. To quantify the hypothetical contribution of each source to the microbial diversity of the 1-year-old lava rock, the number of different genera shared between the lava rock and various environments (soil, air, fumarole, drill core, and seawater) was counted. Genera only found in the 1-year-old lava rocks were considered as an unknown source. The speculative nature of this source prediction method arises from the occurrence of certain genera in multiple environments.

------------------

Reviewer 2: - There are many good ideas, and even literature review in the discussion that really would make

the paper stronger if there was some mention of them in the Introduction.

- In general, I think the authors need to provide more examples/details in this manuscript, rather than simply listing a reference for the reader.

Authors: We believe that the modifications we've implemented in response to the reviewers’ comments will significantly enhance the manuscript by incorporating more details and improving overall quality. In addition, we need to follow the journal policy of limited space and carefully select our text.

**Reviews of manuscript FEMSMC-2023-045.r1**

**Reviewer 2: Rebecca Prescott**

**Recommendation: Minor Revision**

General comments: Thank you for the careful consideration of previous comments from reviewers and incorporating those comments in various ways. The figures are a great improvement to the methods, and understanding the methods used has been improved greatly with the additions and changes. This is a very interesting topic that gets at the potential source and successional questions regarding new lava flows or caves, and how those communities mature and develop over time. You have some very interesting, rare samples.

I do have some questions about the conclusions, importantly about the way the data is presented, particularly in the abstract and maybe even the title. I carefully reviewed the previous comments, responses, and the read the manuscript carefully. From the presentation of the data, and even from the written discussion, it is very clear that the air is likely not the major source for microorganisms on the lava rocks - it is the surrounding soils. I completely agree with the authors that it is likely that some of those organisms in the soils are delivered through the air (so it is likely a conduit). However, the title and abstract suggest to me that the results are that the air is the major source, which is not the case. The abstract does not mention that the surrounding soils contributed a higher percentage than the air. Consistently the results appear to illustrate that the airborne community is quite different than the communities found on the 1 year old and 9 year old rocks. The pattern suggests habitat selection may be occurring from the air populations of microbes, but this is not discussed. This does not mean that the air is not “a” source, but from the data presented, the airborne population appears distinct from the rock, with certain genera being detected from the air? I am still not clear on this from reading the paper. The majority of ASVs are from an unknown source.

I do have some questions about the methods still. In the discussion, the authors state “it appears that the atmosphere has a limited role as a microbial source for the lava rocks. However when we examine the genera-based source prediction….” I am not clear on what the genera-based source prediction is - is from the Source-Tracker? Although additions were made to improve this in the methods, there is not much explanation of it. And it appears that the authors are relying on this one analysis to suggest that the air is a substiental source and a key factor in understanding colonization of microbes on lava flows.

Also, I am very confused about page 6 - line 167 - Comparison of ASVs recovered…

Did the authors only look at ASVs that were found in culture, and not at the entirety of the sequences that were recovered? If so, why? If not, and I have mis-understood, then some clarification is needed in the text.

PERMANOVA was used. Did the data meet the requirements for analysis with PERMANOVA? I ask but it is rare that ecological data or microbial data do, considering that PERMANOVA is based on centroids, and usually ecological data does not meet the assumptions of this analysis. Those need to be provided I think to illustrate the validity of the analysis.

The discussion of this paper is actually very well written, and I think accurately represents the data that is presented. However, adjustments need to be made to the rest of the paper to show this.

There are a number of grammatical mistakes, such as the use of rDNA (incorrect) and the use of rRNA (correct), “et. Al” missing for some references in the paper, and other small mistakes. The authors need to do a careful edit.

I think at times the confusion is coming from a reliance on simply referencing a previous study, rather than providing enough detail for the reader here in this paper. This still needs some improvement.

Response to Responses - Review #2

------------------

Reviewer 2: Line 118-119: did the authors use 16S rRNA sequencing to determine there were no differences in the top, middle, and bottom of the rocks? I would give a little bit more detail here.

Authors: Thank you for bringing this to our attention. Indeed, we used statistical analyses based on the 16S rRNA sequencing to determine the differences in the microbial communities of the different parts of the lava rocks. We added information in the “Material and Method” and “Results” parts for clarity:

In the Material and Method:

Page 5, lines 126-127: The 1-year-old lava rocks were fragmented using a sterilized hammer to facilitate analyses of the top, middle, and bottom parts.

Page 5, line 142: The DNA of the different parts of the rocks and the soil samples were extracted using the DNeasy PowerMax Soil Kit (Qiagen, Hilden, Germany)

In the Results:

Page 7, lines 212-215: Statistical analysis of the separate rock sections revealed only a minor difference in microbial communities between the top and the bottom of the rocks (Table 1). To enhance clarity and align with our goals, the microbial communities from all three sections will be combined and analyzed collectively.

Review 2 response: Looks good overall. I would add the line “To enhance clarity and align with our goals, the microbial communities from all three sections will be combined and analyzed collectively” to the methods as well - or something like it. If its not already there.

————————————————————————————————

Reviewer 2: Line 131: After Lever et al. - should there be a date of publication here?

Authors: Thank you for pointing out this mistake, it was corrected:

Page 5, line 139: The DNA of the air samples was extracted from the Sterivex filters using a modified version of the protocol from Lever and al 2015 ([10], Supplementary Material – Protocol).

Check!

————————————————————————————————-

Reviewer 2: Line 132: can a picture of the tool be provided in Supplemental?

Authors: It is now provided in Supplementary Material – Figure 1.

Thanks looks great.

—————————————————————————————————

Reviewer 2: - For example, there is mention of a fumarole, drill core, and saltwater sample in the discussion, which are not mentioned previously in the paper.

Line 257: where are the fumarole, Drill Core, seawater samples coming from? I believe this is the first mention of them in the manuscript (my apologies if I missed it), but its not clear at the moment while reading it.

Authors: Thank you for these comments. We acknowledge that mentioning these samples earlier in the manuscript will make it clearer. A new sentence was added to the “site description and sample collection” part of the Material and Method:

Page 5, lines 130-132: Additionally, microbial sequences from fumarole, drill core, and seawater samples collected on Surtsey by Bergsten et al. (2021) [9] were incorporated into this study to explore potential sources for the 1-year-old lava rocks of Surtsey.

Are any of the extraction methods different with these samples? Were the samples added, or just the data? I understand that some of this may have been published elsewhere, but its a good idea to be redundant, as people will not go and look up the paper. Your paper needs to stand on its on. So including some details of methods, along with reference to the paper is a good idea.

—————————————————————————————————-

Reviewer 2: - I would also comment that the analysis of the rocks composition should be in the paper, not supplemental. Without these data, its difficult to explain the variation in the samples well, especially with the variation the iron.

Line 289: The composition of the rocks is very important in this study, and should be moved from the Supp. Materials to main body. Along with the methods of that work.

Authors: Based on your comment, we make is more apparent that the compositions were retrieved from the literature and move the Table we made from Supplementary Materials to the main body:

Page 10, lines 298-300: Lava rocks at Fimmvörðuháls contained approximately 8 times more Fe2O3 than the lava rocks of Surtsey (Table 2) (18).

Thank you, important details.

—————————————————————————————————

Reviewer 2: - Same goes for the methods. I don’t know how the samples were collected, what devices were used to collect the air samples, etc.

- I am not clear on how the air samples (or other samples) were collected, and pictures of any air collecting devices would help (same for instruments for the rocks, if any were used).

Authors: We understand the reviewer's concern about the clarity of our sampling methods. Since we are using the same samples as in a previous study and to avoid redundancy and plagiarism, we referred to that publication. However, for better clarity, we added a short paragraph with a brief description of our sampling method, and a picture of our air sampler:

Page 4, lines 117-122: We used air and lava rock samples previously collected as described in our prior publication [7]. Briefly, we employed High-Flow-Rate impingers (Figure 2) for air sample collection, and recovered on-site autoclaved lava rocks after one year, denoted as "1-year-old lava rocks." Additionally, non-autoclaved 9-year-old lava rocks from Fimmvörðuháls were designated as "9-years-old lava rocks". For detailed information on procedures, controls, and other aspects, please consult our previous publication.

That is much more helpful for understanding your project, including the picture.

——————————————————————————————————-

Reviewer 2: - PCoA analysis (Figure 3): The percent variability captured on the x and y axis is quite low (less than 10%), which suggests that this particular metric (Bray-Curtis) or method (PCoA) is not doing a good job of representing the highly complex y-variable data in a 2-dimensional image (something like 30% on PCO 1 suggests the image is representative of the higher dimensional data). Therefore, the image is not meaningful. I would suggest that the authors try using UniFrac metric instead in a PCoA, as it may capture more of the variability and would then be worth including in Figure 3. Otherwise I would remove it from the manuscript as it currently is. Unifrac is available in QIIME2 pipelines, or through EDGE Bioinformatics (which uses the QIIME2 Pipeline). Another option is to use an NMDS rather than PCoA with Bray-Curtis, but suspect you will see the same issue: a high stress value that will show that the graphic is not an acceptable representation of the higher dimensional data. UniFrac usually does a better job at capturing the variability in microbial datasets, so it would be worth trying. Since the authors are relying on this analysis and ordination considerably in their conclusions, I believe this issue needs to be addressed before publication, with at least exploration of other metrics and methods to better assess the groupings of their sampling sites.

Authors: Thank you for this relevant and constructive comment. The PCoA using the UniFrac metric revealed a comparable percentage of variability, with 10.3% on the x-axis and 5.5% on the y-axis. However, the NMDS analysis using UniFrac exhibited a stress value of 0.15, and the NMDS using the Bray-Curtis metric had a slightly lower stress value of 0.14, indicating a more effective representation of the higher dimensional data. Consequently, we have opted to present the NMDS plot using the Bray-Curtis metric in Figure 3, replacing the initial PCoA based on Bray-Curtis. References to the previous PCoA plot were modified in the text to the NMDS.

Great, thank you for making those adjustments. Your stress is still a bit high, but “acceptable” so I would just caution that a “good fit” is usually below 0.1 stress value. And you are slightly above it, so be a bit cautious with your interpretations.——————————————————————————————-

Reviewer 2: Line 236: With the unidentified genera, can you provide a class or order level assessment?

Authors: Our focus with this picture was on highlighting these similar diversity patterns rather than providing specific taxonomic classifications and we intentionally refrained from providing a class or order level assessment. To enhance clarity and prevent confusion, we've opted to remove any reference to these unidentified genera from the text.

———————————————————————————————

Reviewer 2: Line 269: This is the first mention of the HYSPLIT model in the paper, and this is the discussion. Please make sure to add the details of any methods used in the methods, that you want to discuss in the results or Discussion.

Authors: Thank you for pointing this out. To avoid redundancy and plagiarism, we referenced our previous study where the details can be found.

Page 10, lines 279-281: As demonstrated in a previous study [7], the air masses were coming roughly from the same direction in both years and at all sites.

—————————————————————————————-

Reviewer 2: Line 292: were any of these data collected or looked up in meteorological databases/information? If not, this could be very useful information that might help explain the variations you see in samples.

Authors: Due to the remote locations of our sampling sites, we did not have the opportunity to collect or access meteorological data from databases during the current study. However, we acknowledge the significance of meteorological information in understanding variations observed in our samples and therefore modify the text to highlight its potential significance:

Page 10, lines 303-305: Collecting these additional data in a further study would be crucial for a more comprehensive understanding of the factors influencing microbial dynamics and diversity.

Thank you

————————————————————————————

Reviewer 2: Line 295: this is a very interesting comment. Can you connect better to your data? I don’t know what groups you are referring to that have rapid adaptation capabilities? This is an area you could expand on in the Introduction as well - something along the lines of life on rocks and a brief review of that literature.

Authors: Thank you for your feedback. As the groups demonstrating rapid adaptation capabilities will be described later in the manuscript and to maintain clarity in this section, we deleted this part of the sentence.

Following your advice, we have included a few lines in the introduction about colonization:

Page 3, lines 70-73: The process of colonization is influenced by mineral properties, soil pH, and microbial traits. Notably, bacterial binding to minerals, facilitated by extracellular polysaccharides and proteins, is crucial for stable colonization [5], but is notably reduced in acidic and alkaline soils [6].

————————————————————————————

Reviewer 2: Line 246: what is the SourceTracking analysis (its also listed as two words: Source Tracker - decide which way is correct)? I am confused about what this actually is, and it probably needs to be more clearly defined then, and the methods of that written out.

Line 335-336: what is the “genera-based source prediction”? I did a search and this is the only place I can find this phrase. I think you mention looking at the genera-level data earlier in the results, but is not clear if that is what you mean here? Or some other method?

Authors: Thank you for bringing up this concern, addressing this confusion about the methods used to determine the potential sources of the microbes will enhance the overall clarity of the manuscript. The correct term is "SourceTracker," a program specifically designed to forecast the origins of microbial communities within a given set of input samples. To rectify this and better present the “genera-based source prediction”, we have modified a paragraph in the methods section:

Page 6, lines 187-200: Venn diagrams and SourceTracker analyses were used to determine the proportion of soil and air-originated microbes in the 1-year-old lava rocks of Surtsey. Additionally, we employed a method based on genera to explore the potential origin of microbes that didn't come from either air or soil.

The Venn diagram created in Excel shows the common ASVs identified through the microbiome package [13] among the air, the 1-year-old lava rock, and the soil of Surtsey. The SourceTracker package [14], utilizing Bayesian methods [15], assessed the contributions of air and soil to the lava rocks, with each rock individually analyzed, considering air and soil from the same location as potential sources. To quantify the hypothetical contribution of each source to the microbial diversity of the 1-year-old lava rock, the number of different genera shared between the lava rock and various environments (soil, air, fumarole, drill core, and seawater) was counted. Genera only found in the 1-year-old lava rocks were considered as an unknown source. The speculative nature of this source prediction method arises from the occurrence of certain genera in multiple environments.

————————————————————————————

**Editor Decision: Minor Decision**

**Author's Response to Decision Letter for (FEMSMC-2023-045)**

General comments: Thank you for the careful consideration of previous comments from reviewers and incorporating those comments in various ways. The figures are a great improvement to the methods, and understanding the methods used has been improved greatly with the additions and changes. This is a very interesting topic that gets at the potential source and successional questions regarding new lava flows or caves, and how those communities mature and develop over time. You have some very interesting, rare samples.

I do have some questions about the conclusions, importantly about the way the data is presented, particularly in the abstract and maybe even the title. I carefully reviewed the previous comments, responses, and read the manuscript carefully.

Authors: At first, expressing our intended message was challenging because our results were not very straight forward to present. However, our correspondence with the reviewers has definitely made the paper more solid and easier to read. Thank you for your constructive feedback, which will surely enhance the quality of our manuscript.

---------------------------------------------------------------------------------------------------------------------------------------------------------------------

Reviewer: From the presentation of the data, and even from the written discussion, it is very clear that the air is likely not the major source for microorganisms on the lava rocks - it is the surrounding soils. I completely agree with the authors that it is likely that some of those organisms in the soils are delivered through the air (so it is likely a conduit). However, the title and abstract suggest to me that the results are that the air is the major source, which is not the case.

Authors: Thank you for this important comment. The title and part of the abstract were modified to emphasize that the atmosphere serves as a conduit rather than the primary source for bacterial colonization of Icelandic lava rocks:

Title: “Atmospheric dispersal shapes rapid bacterial colonization of Icelandic Lava Rocks”

Abstract: Page 2 lines 45-46: “We hypothesize that the atmosphere serves as a conduit for locally sourced microbes and stress-resistant distant-sourced microbes”

Reviewer: The abstract does not mention that the surrounding soils contributed a higher percentage than the air. Consistently the results appear to illustrate that the airborne community is quite different than the communities found on the 1-year-old and 9-year-old rocks.

Authors: Thank you for pointing this out, we modified the abstract to include this finding:

Abstract: page 2 lines 39-41: “SourceTracker analysis revealed distinct bacterial populations in the atmosphere and the lava rocks with surrounding soil contributed more significantly to lava rock microbial composition.”

---------------------------------------------------------------------------------------------------------------------------------------------------------------------

Reviewer: The pattern suggests habitat selection may be occurring from the air populations of microbes, but this is not discussed.

Authors: We added a sentence in the manuscript to address this point:

Page 12 lines 357-359: “Furthermore, the significant exchange observed between soil and rock, alongside the shared genera between lava rocks and other environments, including air samples, indicates that habitat selection may be occurring from the air populations of microbes.”

Reviewer: This does not mean that the air is not “a” source, but from the data presented, the airborne population appears distinct from the rock, with certain genera being detected from the air? I am still not clear on this from reading the paper. The majority of ASVs are from an unknown source.

Authors: In our study, we initially focused on analyzing the amplicon sequence variants (ASVs) from lava rocks, and interestingly, the majority of these variants did not appear to originate from either soil or air sources. In an additional exploratory analysis, our findings revealed that certain genera were present in both the air and the rocks, suggesting their potential to thrive and be exchanged between these environments. This observation supports the notion that certain microbes can survive in diverse habitats and may undergo exchange or dispersal between these environments. This will be clarified by answering the next remark.

---------------------------------------------------------------------------------------------------------------------------------------------------------------------

Reviewer: I do have some questions about the methods still. In the discussion, the authors state “it appears that the atmosphere has a limited role as a microbial source for the lava rocks. However when we examine the genera-based source prediction….”

I am not clear on what the genera-based source prediction is - is from the Source-Tracker? Although additions were made to improve this in the methods, there is not much explanation of it. And it appears that the authors are relying on this one analysis to suggest that the air is a substiental source and a key factor in understanding colonization of microbes on lava flows.

Authors: We understand the confusion and have revised the manuscript to provide a clearer explanation of the method, along with its justification and acknowledgment of its speculative nature. Additionally, we have refrained from labelling it as a "genera-based method" to avoid any ambiguity.

In the abstract: page 2 lines 41-42:

“Nevertheless, shared genera among air, rocks, and local sources, suggested potential exchange between these environments.”

In the methods: page 7 lines 197-202: “To quantify the hypothetical contribution of each source to the microbial diversity of the 1-year-old lava rock, we performed a supplementary investigation in which we compared the presence of genera across the lava rocks and different environments (soil, air, fumarole, drill core, and seawater), suggesting potential dispersal between them. The speculative nature of this source prediction method arises from the occurrence of certain genera in multiple environments.”

In the discussion: page 12 lines 341-345: “Moreover, we discovered that a significant proportion (83 %) of the genera identified on the 1-year-old lava rocks at Surtsey were also found in the other investigated environments, suggesting potential exchange dynamics between them. Notably, an average of 71% of the genera present in the 1-year-old lava rock and other investigated sources were also detected in the air samples (as shown in Supplementary Material: Figure 6).”

---------------------------------------------------------------------------------------------------------------------------------------------------------------------

Reviewer: Also, I am very confused about page 6 - line 167 - Comparison of ASVs recovered… Did the authors only look at ASVs that were found in culture, and not at the entirety of the sequences that were recovered? If so, why? If not, and I have mis-understood, then some clarification is needed in the text.

Authors: In this study, we analyzed all the ASVs obtained from our samples. However, when comparing the ASVs recovered by both culture and amplicon methods, we restricted our analysis to only include the samples that were common to both approaches. We understand the confusion and reformulated the sentence for clarity.

Page 6 lines 162-163: “To compare the ASVs recovered from both cultivation [9] and amplicon sequencing, only the samples utilized in both methods were considered.”

---------------------------------------------------------------------------------------------------------------------------------------------------------------------

Reviewer: PERMANOVA was used. Did the data meet the requirements for analysis with PERMANOVA? I ask but it is rare that ecological data or microbial data do, considering that PERMANOVA is based on centroids, and usually ecological data does not meet the assumptions of this analysis. Those need to be provided I think to illustrate the validity of the analysis.

Authors: Thank you for your comment. We first conducted a PERMDISP test to evaluate dispersion differences among the groups, followed by a PERMANOVA test to assess differences in group means. Both analyses revealed statistically significant differences, strengthening the reliability of our findings. Furthermore, we conducted a post-hoc Tukey's Honest Significant Differences (Tukey’s HSD) test to further validate these differences. Our NMDS plot corroborates these findings, displaying distinct differences in both mean and dispersion among the groups between the groups. We added a paragraph to the methods to mention these tests:

Page 7 lines 187-191: “Before executing the PERMANOVA analysis, we ensured the prerequisites were met by conducting pre-assessments using PERMDISP and Tukey's Honest Significant Differences tests (Tukey’s HSD). These evaluations confirmed significant statistical disparities in both dispersion between groups and groups means, thereby supporting the validity of our findings (Supplementary material – Table 2).”

---------------------------------------------------------------------------------------------------------------------------------------------------------------------

Reviewer: The discussion of this paper is actually very well written, and I think accurately represents the data that is presented. However, adjustments need to be made to the rest of the paper to show this.

There are a number of grammatical mistakes, such as the use of rDNA (incorrect) and the use of rRNA (correct), “et. Al” missing for some references in the paper, and other small mistakes. The authors need to do a careful edit.

Author: Thank you for pointing it out to us, we carefully checked the manuscript for any grammatical mistakes.

Reviewer: I think at times the confusion is coming from a reliance on simply referencing a previous study, rather than providing enough detail for the reader here in this paper. This still needs some improvement.

---------------------------------------------------------------------------------------------------------------------------------------------------------------------

---------------------------------------------------------------------------------------------------------------------------------------------------------------------

Response to Response to Responses - Review #2

Reviewer 2: Line 118-119: did the authors use 16S rRNA sequencing to determine there were no differences in the top, middle, and bottom of the rocks? I would give a little bit more detail here.

Authors: Thank you for bringing this to our attention. Indeed, we used statistical analyses based on the 16S rRNA sequencing to determine the differences in the microbial communities of the different parts of the lava rocks. We added information in the “Material and Method” and “Results” parts for clarity: In the Material and Method: Page 5, lines 126-127: The 1-year-old lava rocks were fragmented using a sterilized hammer to facilitate analyses of the top, middle, and bottom parts. Page 5, line 142: The DNA of the different parts of the rocks and the soil samples were extracted using the DNeasy PowerMax Soil Kit (Qiagen, Hilden, Germany) In the Results: Page 7, lines 212-215: Statistical analysis of the separate rock sections revealed only a minor difference in microbial communities between the top and the bottom of the rocks (Table 1). To enhance clarity and align with our goals, the microbial communities from all three sections will be combined and analyzed collectively.

Review 2 response: Looks good overall. I would add the line “To enhance clarity and align with our goals, the microbial communities from all three sections will be combined and analyzed collectively” to the methods as well - or something like it. If its not already there.

Authors response: Thank you. This line was added:

Page 3 lines 126-128: “Based on the first results and to enhance clarity and align with our goals, the microbial communities from all three sections will be combined and analyzed collectively.”

----------------------------------------------------------------------------------------

Reviewer 2: Line 131: After Lever et al. - should there be a date of publication here?

Authors: Thank you for pointing out this mistake, it was corrected: Page 5, line 139: The DNA of the air samples was extracted from the Sterivex filters using a modified version of the protocol from Lever and al 2015 ([10], Supplementary Material – Protocol).

Review 2 response: Check!

----------------------------------------------------------------------------------------

Reviewer 2: Line 132: can a picture of the tool be provided in Supplemental?

Authors: It is now provided in Supplementary Material – Figure 1.

Review 2 response: Thanks looks great.

----------------------------------------------------------------------------------------

Reviewer 2: - For example, there is mention of a fumarole, drill core, and saltwater sample in the discussion, which are not mentioned previously in the paper. Line 257: where are the fumarole, Drill Core, seawater samples coming from? I believe this is the first mention of them in the manuscript (my apologies if I missed it), but its not clear at the moment while reading it.

Authors: Thank you for these comments. We acknowledge that mentioning these samples earlier in the manuscript will make it clearer. A new sentence was added to the “site description and sample collection” part of the Material and Method: Page 5, lines 130-132: Additionally, microbial sequences from fumarole, drill core, and seawater samples collected on Surtsey by Bergsten et al. (2021) [9] were incorporated into this study to explore potential sources for the 1-year-old lava rocks of Surtsey.

Review 2 response: Are any of the extraction methods different with these samples? Were the samples added, or just the data? I understand that some of this may have been published elsewhere, but its a good idea to be redundant, as people will not go and look up the paper. Your paper needs to stand on its on. So including some details of methods, along with reference to the paper is a good idea.

Authors response: Thank you for this comment, only the data were added and we modified the manuscript to enhance its clarity:

Page 4 lines 131-133: “Additionally, 16S rRNA sequences from fumarole, drill core, and seawater samples collected on Surtsey by Bergsten et al. (2021) [11] were incorporated into this study to explore potential sources for the 1-year-old lava rocks of Surtsey.”

----------------------------------------------------------------------------------------

Reviewer 2: - I would also comment that the analysis of the rocks composition should be in the paper, not supplemental. Without these data, its difficult to explain the variation in the samples well, especially with the variation the iron. Line 289: The composition of the rocks is very important in this study, and should be moved from the Supp. Materials to main body. Along with the methods of that work.

Authors: Based on your comment, we make is more apparent that the compositions were retrieved from the literature and move the Table we made from Supplementary Materials to the main body: Page 10, lines 298-300: Lava rocks at Fimmvörðuháls contained approximately 8 times more Fe2O3 than the lava rocks of Surtsey (Table 2) (18).

Review 2 response: Thank you, important details.

----------------------------------------------------------------------------------------

Reviewer 2: - Same goes for the methods. I don’t know how the samples were collected, what devices were used to collect the air samples, etc. - I am not clear on how the air samples (or other samples) were collected, and pictures of any air collecting devices would help (same for instruments for the rocks, if any were used).

Authors: We understand the reviewer's concern about the clarity of our sampling methods. Since we are using the same samples as in a previous study and to avoid redundancy and plagiarism, we referred to that publication. However, for better clarity, we added a short paragraph with a brief description of our sampling method, and a picture of our air sampler: Page 4, lines 117-122: We used air and lava rock samples previously collected as described in our prior publication [7]. Briefly, we employed High-Flow-Rate impingers (Figure 2) for air sample collection, and recovered on-site autoclaved lava rocks after one year, denoted as "1-year-old lava rocks." Additionally, non-autoclaved 9-year-old lava rocks from Fimmvörðuháls were designated as "9-years-old lava rocks". For detailed information on procedures, controls, and other aspects, please consult our previous publication.

Review 2 response: That is much more helpful for understanding your project, including the picture.

----------------------------------------------------------------------------------------

Reviewer 2: - PCoA analysis (Figure 3): The percent variability captured on the x and y axis is quite low (less than 10%), which suggests that this particular metric (Bray-Curtis) or method (PCoA) is not doing a good job of representing the highly complex y-variable data in a 2-dimensional image (something like 30% on PCO 1 suggests the image is representative of the higher dimensional data). Therefore, the image is not meaningful. I would suggest that the authors try using UniFrac metric instead in a PCoA, as it may capture more of the variability and would then be worth including in Figure 3. Otherwise I would remove it from the manuscript as it currently is. Unifrac is available in QIIME2 pipelines, or through EDGE Bioinformatics (which uses the QIIME2 Pipeline). Another option is to use an NMDS rather than PCoA with Bray-Curtis, but suspect you will see the same issue: a high stress value that will show that the graphic is not an acceptable representation of the higher dimensional data. UniFrac usually does a better job at capturing the variability in microbial datasets, so it would be worth trying. Since the authors are relying on this analysis and ordination considerably in their conclusions, I believe this issue needs to be addressed before publication, with at least an exploration of other metrics and methods to better assess the groupings of their sampling sites.

Authors: Thank you for this relevant and constructive comment. The PCoA using the UniFrac metric revealed a comparable percentage of variability, with 10.3% on the x-axis and 5.5% on the y-axis. However, the NMDS analysis using UniFrac exhibited a stress value of 0.15, and the NMDS using the Bray-Curtis metric had a slightly lower stress value of 0.14, indicating a more effective representation of the higher dimensional data. Consequently, we have opted to present the NMDS plot using the BrayCurtis metric in Figure 3, replacing the initial PCoA based on Bray-Curtis. References to the previous PCoA plot were modified in the text to the NMDS.

Review 2 response: Great, thank you for making those adjustments. Your stress is still a bit high, but “acceptable” so I would just caution that a “good fit” is usually below 0.1 stress value. And you are slightly above it, so be a bit cautious with your interpretations.

Authors response: Thank you for your advice, we added a sentence in the manuscript to address it:

Page 8 lines 218-221: “The Bray-Curtis-based non-metric multidimensional scaling (NMDS) exhibited a stress value of 0.14, indicating the need for caution in interpreting the results. However, notable trends are clearly apparent and showed distinct microbial communities between the air and the 1-year-old lava rock samples (Figure 4).”

----------------------------------------------------------------------------------------

Reviewer 2: Line 236: With the unidentified genera, can you provide a class or order level assessment?

Authors: Our focus with this picture was on highlighting these similar diversity patterns rather than providing specific taxonomic classifications and we intentionally refrained from providing a class or order level assessment. To enhance clarity and prevent confusion, we've opted to remove any reference to these unidentified genera from the text.

----------------------------------------------------------------------------------------

Reviewer 2: Line 269: This is the first mention of the HYSPLIT model in the paper, and this is the discussion. Please make sure to add the details of any methods used in the methods, that you want to discuss in the results or Discussion.

Authors: Thank you for pointing this out. To avoid redundancy and plagiarism, we referenced our previous study where the details can be found. Page 10, lines 279-281: As demonstrated in a previous study [7], the air masses were coming roughly from the same direction in both years and at all sites.

----------------------------------------------------------------------------------------

Reviewer 2: Line 292: were any of these data collected or looked up in meteorological databases/information? If not, this could be very useful information that might help explain the variations you see in samples.

Authors: Due to the remote locations of our sampling sites, we did not have the opportunity to collect or access meteorological data from databases during the current study. However, we acknowledge the significance of meteorological information in understanding variations observed in our samples and therefore modify the text to highlight its potential significance: Page 10, lines 303-305: Collecting these additional data in a further study would be crucial for a more comprehensive understanding of the factors influencing microbial dynamics and diversity.

Review 2 response: Thank you

----------------------------------------------------------------------------------------

Reviewer 2: Line 295: this is a very interesting comment. Can you connect better to your data? I don’t know what groups you are referring to that have rapid adaptation capabilities? This is an area you could expand on in the Introduction as well - something along the lines of life on rocks and a brief review of that literature.

Authors: Thank you for your feedback. As the groups demonstrating rapid adaptation capabilities will be described later in the manuscript and to maintain clarity in this section, we deleted this part of the sentence. Following your advice, we have included a few lines in the introduction about colonization: Page 3, lines 70-73: The process of colonization is influenced by mineral properties, soil pH, and microbial traits. Notably, bacterial binding to minerals, facilitated by extracellular polysaccharides and proteins, is crucial for stable colonization [5], but is notably reduced in acidic and alkaline soils [6].

----------------------------------------------------------------------------------------

Reviewer 2: Line 246: what is the SourceTracking analysis (its also listed as two words: Source Tracker - decide which way is correct)? I am confused about what this actually is, and it probably needs to be more clearly defined then, and the methods of that written out. Line 335-336: what is the “genera-based source prediction”? I did a search and this is the only place I can find this phrase. I think you mention looking at the genera-level data earlier in the results, but is not clear if that is what you mean here? Or some other method?

Authors: Thank you for bringing up this concern, addressing this confusion about the methods used to determine the potential sources of the microbes will enhance the overall clarity of the manuscript. The correct term is "SourceTracker," a program specifically designed to forecast the origins of microbial communities within a given set of input samples. To rectify this and better present the “genera-based source prediction”, we have modified a paragraph in the methods section: Page 6, lines 187-200: Venn diagrams and SourceTracker analyses were used to determine the proportion of soil and air-originated microbes in the 1-year-old lava rocks of Surtsey. Additionally, we employed a method based on genera to explore the potential origin of microbes that didn't come from either air or soil. The Venn diagram created in Excel shows the common ASVs identified through the microbiome package [13] among the air, the 1-year-old lava rock, and the soil of Surtsey. The SourceTracker package [14], utilizing Bayesian methods [15], assessed the contributions of air and soil to the lava rocks, with each rock individually analyzed, considering air and soil from the same location as potential sources. To quantify the hypothetical contribution of each source to the microbial diversity of the 1-year-old lava rock, the number of different genera shared between the lava rock and various environments (soil, air, fumarole, drill core, and seawater) was counted. Genera only found in the 1-year-old lava rocks were considered as an unknown source. The speculative nature of this source prediction method arises from the occurrence of certain genera in multiple environments.

**Reviews of manuscript FEMSMC-2023-045.r2**

**Reviewer 2: Rebecca Prescott**

**Recommendation: Accept**

No additional changes are necessary, some minor grammatical corrections could be made before publication.

**Editor Decision: Accept**
